# Comparative analysis of infected cassava root transcriptomics reveals candidate genes for root rot disease resistance

**DOI:** 10.1038/s41598-024-60847-4

**Published:** 2024-05-08

**Authors:** Camila Santiago Hohenfeld, Saulo Alves Santos de Oliveira, Claudia Fortes Ferreira, Victor Hugo Mello, Gabriel Rodrigues Alves Margarido, Adriana Rodrigues Passos, Eder Jorge de Oliveira

**Affiliations:** 1 Universidade Estadual de Feira de Santana, Av. Transnordestina, S/N - 44036-900, Novo Horizonte, Feira de Santana, BA Brazil; 2 Embrapa Mandioca e Fruticultura, Rua da Embrapa, Caixa Postal 007, Cruz das Almas, BA 44380-000 Brazil; 3 Departamento de Genética, Escola Superior de Agricultura “Luiz de Queiroz”, Universidade de São Paulo, Avenida Pádua Dias, 11, Piracicaba, SP 13418-900 Brazil

**Keywords:** Breeding, *Manihot esculenta* Crantz, Soil-borne diseases, Resistant cultivar, Biotechnology, Genetics, Molecular biology, Plant sciences

## Abstract

Cassava root-rot incited by soil-borne pathogens is one of the major diseases that reduces root yield. Although the use of resistant cultivars is the most effective method of management, the genetic basis for root-rot resistance remains poorly understood. Therefore, our work analyzed the transcriptome of two contrasting genotypes (BRS Kiriris/resistant and BGM-1345/susceptible) using RNA-Seq to understand the molecular response and identify candidate genes for resistance. Cassava seedlings (resistant and susceptible to root-rot) were both planted in infested and sterilized soil and samples from Initial-time and Final-time periods, pooled. Two controls were used: (i) seedlings collected before planting in infested soil (absolute control) and, (ii) plants grown in sterilized soil (mock treatments). For the differentially expressed genes (DEGs) analysis 23.912 were expressed in the resistant genotype, where 10.307 were differentially expressed in the control treatment, 15 DEGs in the Initial Time-period and 366 DEGs in the Final Time-period. Eighteen candidate genes from the resistant genotype were related to plant defense, such as the MLP-like protein 31 and the peroxidase A2-like gene. This is the first model of resistance at the transcriptional level proposed for the cassava × root-rot pathosystem. Gene validation will contribute to screening for resistance of germplasm, segregating populations and/or use in gene editing in the pursuit to develop most promising cassava clones with resistance to root-rot.

## Introduction

Ranked by the United Nations as the most important staple food crop of the twenty-first century, cassava (*Manihot esculenta* Crantz) is mostly cultivated by smallholders in developing countries in Africa, Asia and Latin America^1^. Cassava is widely cultivated in Brazil, being the fourth largest producer worldwide, with 18 million tons of cassava roots produced annually over an area of 1.24 million hectares. As it is of Brazilian origin, there are more than 150 local varieties used in the country, whose cycles and production systems vary greatly due to the different environmental conditions that occur from north to south of Brazil. Its economic and social relevance relies on the ability of cassavas to thrive in marginal soils and water deficit conditions, whereas other crops would fail considerably. Cassavas adapt easily contributing to alleviate poverty and its multiple use of starch has great appeal and interest by the agribusiness and industry sectors^2^. However, fungal, bacterial and viral diseases are among the biotic factors that most limit cassava production. The losses from diseases are not just an agricultural problem, but represent a serious threat to food security and economic development in cassava producing countries^3^.

Root rot diseases directly affect the main commercial product of cassava which is the starch-rich tuberous root and can causes losses up to 100%^4^. Caused by soil-borne pathogens, it drastically reduces the quality and productivity of roots and may even make the reuse of areas unfeasible due to the accumulation of primary inoculum of the pathogens involved in the complex^4^. Cassava root rot diseases (CRRD) are caused by a complex of different species of phytopathogens and the symptoms may vary as follows: (i) soft root rot: caused by oomycetes of the *Phytophthora*, *Pythium* and *Phytopythium* genera; (ii) dry root rot: caused by fungi belonging to the genus Fusarium and to the species *Macrophomina pseudophaseolina*; (iii) and black root rot: associated with species of the Botryosphareacea family^5,6^. These phytopathogens are difficult to control since many of them can produce resistance structures such as chlamydospores that allow their survival in the soil for several years in the absence of a host, as well as, the major cassava varieties can be affected by the disease in any growth stage^7^.

Diseases incited by soil-borne pathogens are a serious problem for agriculture due to the complexity of their interaction with the environment, difficult management and control^8^. Different management strategies have been reported for CRRD such as crop rotation, chemical control, soil disinfestation by water vapor, soil solarization, alternative controls by plant extracts and oils, and use of biological control agents^7,9–12^. However, the use of genetic resistance is considered the less expensive and most environmentally friendly method for cassava root rot disease management in the long term^13–16^.

Inoculations in detached roots in addition to inoculations of stems and leaves are the main strategies for initial screening and identification of sources of resistance to root rot disease in cassava. However, more recent studies show that there are differences between resistance mechanisms in different plant tissues^16–18^. A putative explanation is that resistance to root rot may have a quantitative pattern of inheritance controlled by minor genes characterized by the absence of complete or qualitative resistance^17^. Therefore, the chance of success in obtaining cultivars resistant to this disease is directly linked to the knowledge of the genetic aspects related to the interactions with this pathosystem^19^.

Recent advances in plant physiology, biochemistry and genomics have contributed to the understanding of how plants respond to biotic stresses and which mechanisms are responsible for the observed differences in resistances^20^. Among these advances, total RNA sequencing (RNA-Seq) has been a revolutionary tool in transcriptomics as it allows the entire transcriptome to be assessed in a quantitative manner with accurate detection of variable expression levels under different stress conditions^21^.

The availability of the cassava reference genome^22^ provided new opportunities for the characterization of the transcriptome related to responses to environmental stimuli and stresses, as well as cassava diseases such as anthracnose, bacterial blight and cassava brown streak disease^23–25^. However, there are no studies available on the response of cassava genotypes to mixed infection by pathogens that cause black and dry root rot diseases at the transcriptional level.

The use of biotechnological tools to study this pathosystem in depth is lacking. The few studies developed to date have focused on Genome Wide Association Studies—GWAS^17,26^ and semi-quantitative analyses by RT-PCR^27^, which identified possible genes involved in defense mechanisms to root-rot. However, in order to understand the defense mechanisms in cassava against root rot, a more thorough evaluation is necessary to better understand the genetic pathway of resistance and corroborate epidemiological studies. Therefore, the objective of this work was to analyze the transcriptome of two contrasting genotypes of cassava in response to mixed infection by the fungal complex that causes dry and black root-rot, as well as to understand the response of the most resistant genotype in two time-points of interaction in order to identify possible root-rot resistance candidate genes.

## Results

The method of seedlings inoculation in infested soil resulted in characteristic symptoms of the disease for the BGM-1345 (susceptible) cultivar, such as wilting and yellowing of the leaves, in an average period of 20 days after inoculation (DAI) (Fig. S1), while the foliar symptoms were not present in the BRS Kiriris cultivar. As a result, the BGM-1345 genotype died at 30 DAI, while the controls did not show any symptoms of root rot. In all evaluations, the isolation of pathogens that caused root rot was possible from tissues of the seedlings transplanted in infested soil, completing Koch's postulates. Differences in root colonization between varieties and treatments were identified by the method of clarification of root fragments and staining of the fungal structures with Trypan blue.

**Figure 1:**
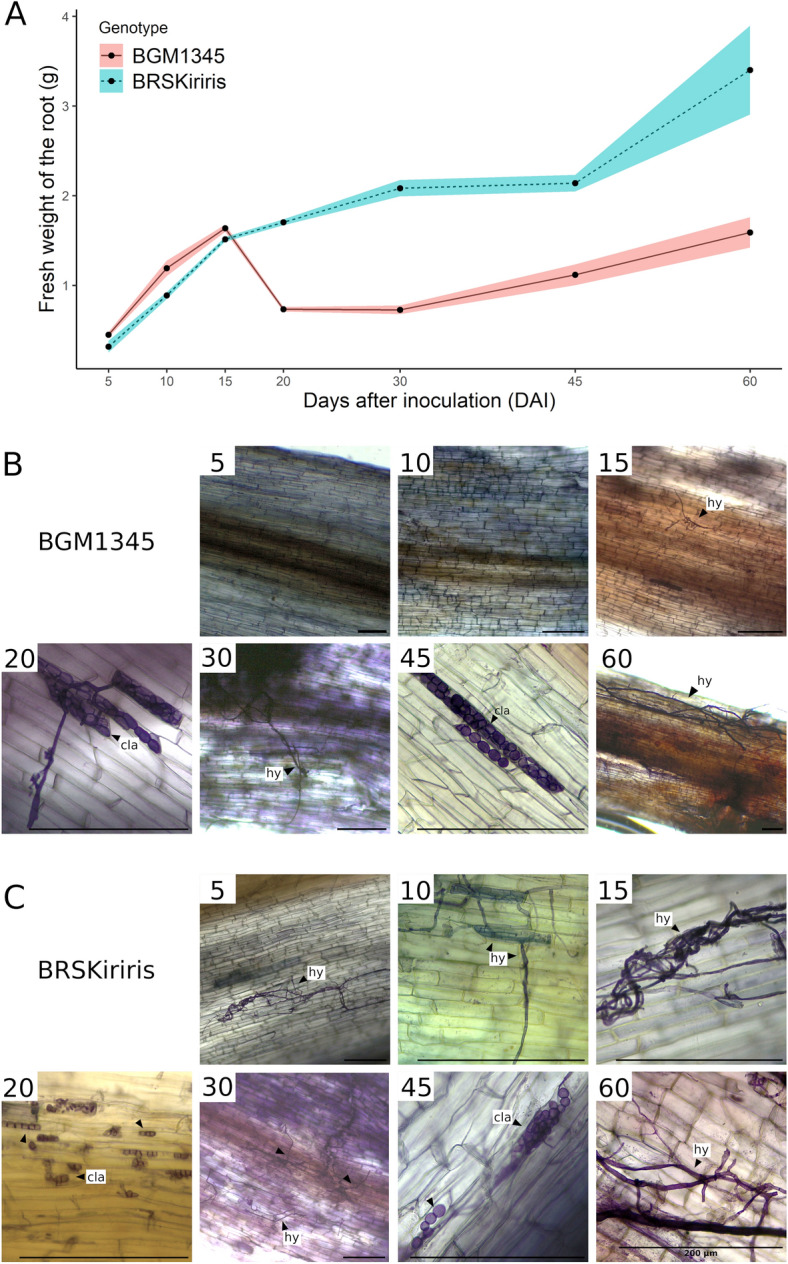
(**A**) Fresh root weight (g) of resistant (BRS Kiriris) and susceptible (BGM-1345) cassava genotypes grown in soil infested with root rot pathogens between 5 and 60 days after planting. (**B**) Clarification of root fragments and staining of fungal structures of the cassava genotype BGM-1345 at seven collection time-periods after planting in infested soil (5, 10, 15, 20, 30, 45, 60 days after planting). (**C**) Clarification of root fragments and staining of fungal structures of cassava genotype BRS Kiriris at seven collection time-periods after planting in infested soil (5, 10, 15, 20, 30, 45, 60 days after planting). hy = hyphae; cla: chlamydospore.

No fungal structures were detected in the roots of the two varieties in the non-inoculated (absolute control) and in the ‘Initial time 0’, indicating that the transplanted plants were healthy (without any colonization by pathogens) (Fig. S1). Fungal structures were also not found in roots of the two varieties collected in non-infested soil. However, root fragments of the variety BRS Kiriris (resistant) in infested soil showed colonization by root rot pathogens, evidenced by the presence of septate hyphae and chlamydospores at all seven collection times (Fig. 1A), which indicates that the variety does not have complete resistance to the disease (immunity profile). Regarding the genotype BGM-1345 (susceptible) the staining of roots did not demonstrate pathogen structures in the two initial collection time-periods (5 and 10 days), although pathogen structures (chlamydospores and hyphae) were identified at 15, 20, 30, 45 and 60 days after planting in infested soil (Fig. 1B).

Differences were also identified between cassava genotypes as to fresh root weight (g) across collection time-periods (Fig. 1C). In the initial period, up to 15 DAI in infested soil, the genotypes did not differ as to root weight. After 20 days, BGM-1345 showed a significant reduction of the root system, while the variety BRS Kiriris remained with constant growth of the root system. It is likely that the mortality of the roots of the susceptible genotype from 15 days onwards is a result of the infection process caused by the pathogens. A growth resumption cycle in the susceptible variety that survived, occurred slowly only after 45 DAI.

### Analysis of transcriptomic profile via RNA-Seq

Sequencing of the 20 libraries generated a total of 1,012,940,297 reads (Table S1) and 510,218,500 quality-filtered and trimmed reads were suitable for the assembly of transcript sequences. These filtered reads were aligned with gene models and reference transcripts from cassava. In general, the gene distribution showed that most of the reads (mean 69.37%) were mapped to coding regions, another 11.87% to intergenic transcripts and 18.73% to introns.

The number of genes aligned differed between the two genotypes. For the susceptible genotype (BGM-1345) 29,479,831 aligned fragments were aligned under normal planting conditions (absolute control), 20,728,585 aligned fragments at the initial time-period of interaction after planting in infested soil, and 20,154,412 aligned fragments at the final time-period of interaction. While for the resistant genotype, 34,594,850 aligned fragments were reported in the normal planting condition, 25,194,515 aligned fragments in the initial time-period and 26,459,295 aligned fragments in the final time-period. On average, 50.16% of the reads were mapped to the cassava reference transcripts.

### Differential gene expression

**Figure 2:**
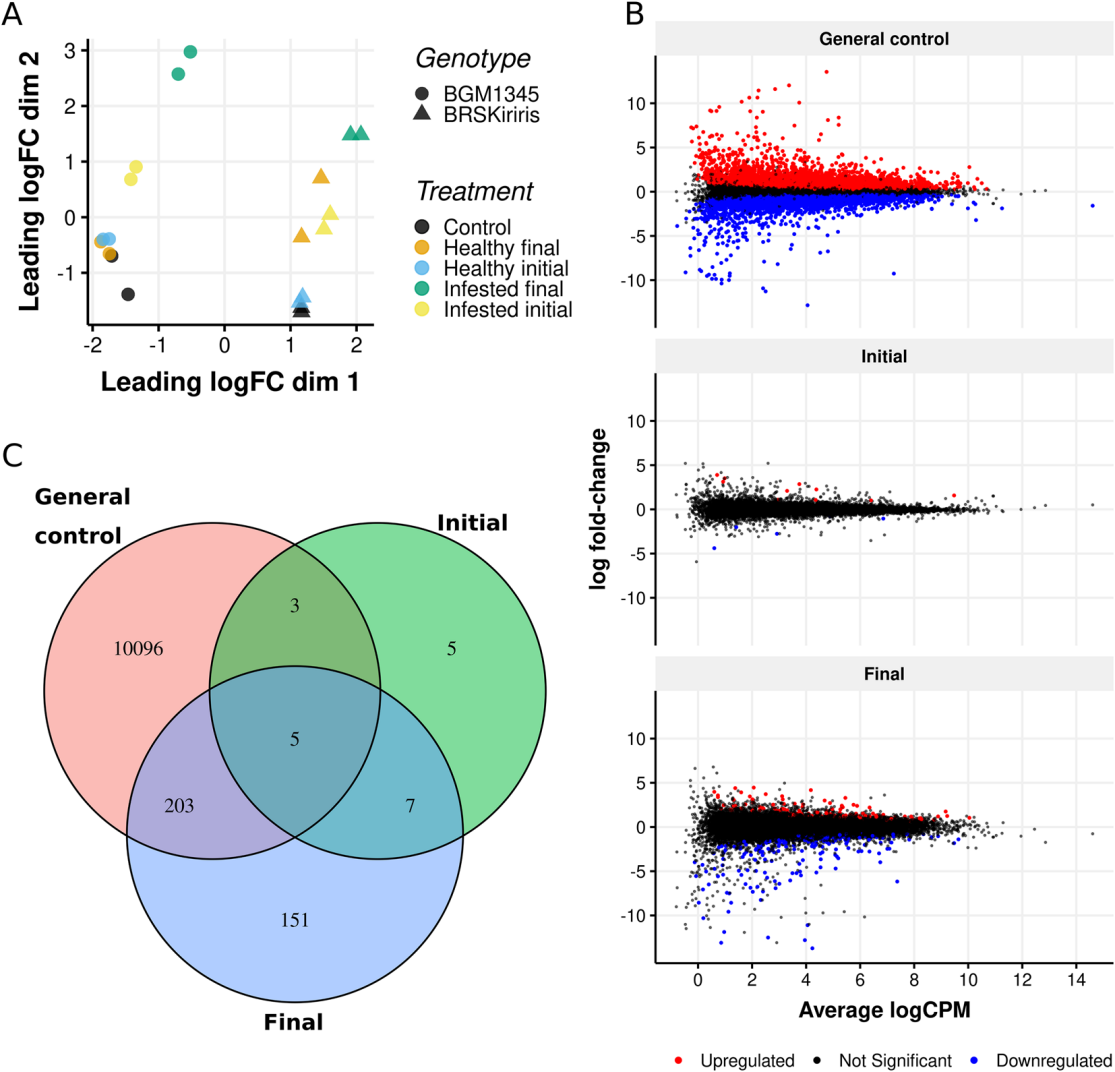
(**A**) Multidimensional scaling plot (MDSPlot) based on logFC (Fold Change) values generated by counting the reads of BRS Kiriris and BGM-1345 genotypes, inoculated with pathogens associated with root rot. (**B**) Mean-difference plot (MD-Plot) based on differential gene expression tests at each time-period for the contrast between BRS Kiriris × BGM-1345. *Up-regulated* indicates the genes of increased expression and *Down-regulated* indicates repressed genes in BRS Kiriris, *Non-DE* = not differentially expressed. (**C**) Venn diagram with differentially expressed transcripts (*p-value* < *0.05*) of the interaction between cassava genotypes *versus* root rot for each of the treatments (Control, Initial time-period and Final time-period). Absolute numbers represent differentially expressed transcripts that are unique or common among pairs or among all treatments.

Genes identified in low frequency were eliminated from the differential expression analysis in order to avoid incorporation of sequencing errors or low reliability information. Therefore, genes with at least two counts per million (CPM), in each sample, were selected, resulting in 23,912 genes kept for the next steps. After normalization of the read counts, the results were visually verified based on a multidimensional scaling representing the distances in expression profiles between all samples. Thus, the general pattern of transcription in each replicate was identified as well as the relationship between treatments and genotypes (Fig. 2A). The first dimension of the graph separated very clearly the two genotypes evaluated, regardless of inoculation, indicating a large biological variation between their transcripts. Possibly the response of the two genotypes to the penetration and colonization of root rot pathogens has a direct effect on this basal difference in transcriptional profile since it is a case of resistance (BRS Kiriris) and extreme susceptibility (BGM-1345).

Regarding inoculation conditions to which the experiment was conducted, in general, the two replicates of each treatment remained close, indicating that there was very little biological variance between the samples sequenced for the same condition. Conversely, sampling in time (Initial time-period × Final time-period) and planting condition (Infested Soil × Healthy Soil) showed wide variation in the transcriptional profile (Fig. 2A). The treatments without inoculation (“Control”, “Healthy-Initial time-period”, “Healthy-Final time-period”) showed an overlap for the BGM-1345 genotype, as an indication of less variance between these groups. Thus, the time-period did not interfere in the transcriptional profile of the susceptible genotype. In contrast, there was also an overlap of the treatments "Control" and "Healthy-Initial time-period" for the BRS Kiriris genotype, although there was a greater distance from the treatment "Healthy-Final time-period" indicating an effect of time on the gene expression of this genotype.

For both genotypes, samples belonging to the “Infested-Final time-period” group showed the largest distance in comparison to the others, confirming the transcriptional effect of the pathogen inoculation process. This separation, visible in the second dimension of the graph, indicates that there was a larger difference in gene expression between this treatment and the others, providing evidence that these samples are biologically more discrepant (Fig. 2A).

### Differentially expressed genes

Among the 23,912 genes analyzed, 10,307 were differentially expressed (DE) in the “Control” treatment, with 5165 repressed and 5,142 activated in the resistant genotype compared to the susceptible one. For the “Initial Time-period” treatment, there were only 15 DEGs, five of which were repressed and ten activated, while in the “Final Time-period” treatment, 366 DE genes were detected, of which 235 were repressed and 131 activated (Fig. 2B and Table 1).

**Table 1:** Results of differential expression tests for the contrast between BRS Kiriris and BGM-1345 for all treatments.

Category	Number of genes		
	General control	Initial time-period	Final time-period
Upregulated	5.142	10	131
Downregulated	5.165	5	235
Non-significant	13.605	23.897	23.546
Total differentially expressed	10.307	15	366
Total genes	23.912		

The distribution of the DEGs is illustrated in the Venn diagram (Fig. 2C). Of the 23.912 DEGs, five were common for all three conditions, three were shared between 'Control' and 'Initial Time-period', seven genes are shared between the 'Initial Time-period and 'Final Time-period' and 203 genes were shared between 'Control' and 'FinalTime-period'; in addition to 10.096 being exclusive to the 'Control' treatment; five to the 'Initial Time-period'; and 151 to the 'Final Time-period' treatment (Fig. 2C).

### Functional assessment of differentially expressed genes

For a better understanding of metabolic alterations in each condition, functional annotations of the differentially expressed genes, were performed. Several general aspects regarding cassava genotypes considered resistant to root-rot under normal planting conditions (Absolute Control) and the changes caused by inoculation (planting in infested soil) at the two different interaction times (Initial and final time-periods), were identified.

**Figure 3:**
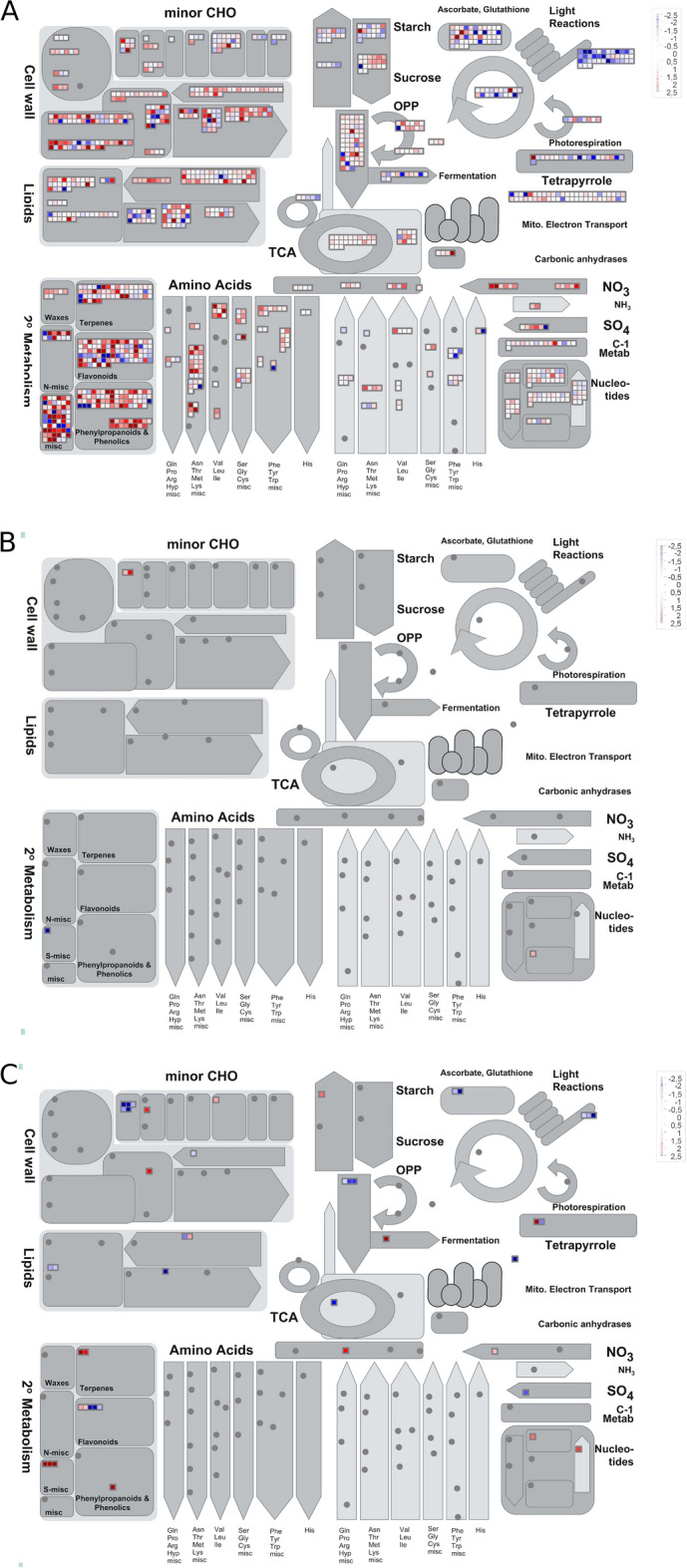
General overview of cassava metabolism based on the *MapMan* software from logFC (*FoldChange*) in treatment “Control” (**A**); “Initial Time-period” (**B**) and “Final time-period” (**C**). Each square in the figure represents a different gene mapped to the same metabolic process. Blue, red, and white colors represent down-regulated, up-regulated and non-differentially regulated genes, respectively.

For the “Absolute Control” (Fig. 3A) 1,007 genes (9.77%) were reported from a total of 10,307 mapped DEGs. Another three genes (20%) out of a total of 15 mapped in the “Initial Time-period” condition (Fig. 3B) and 36 genes (9.83%) out of 366 mapped in the “Final Time-period” condition (Fig. 3C), are also shown.

Root rot infection probably promoted changes in the cassava metabolic pathway, since different profiles of the transcripts were found for the before and after inoculation time-periods. At the initial time-period of the interaction, only two metabolic pathways presented differentially expressed genes, the metabolism of smaller carbohydrate molecules (*minorCHO*), related to the metabolism of compounds associated with the cell wall and nucleotides. All were up-regulated. In contrast, for the final time-period of the interaction, the processes involved with the up-regulated DEGs were associated with the cell wall (metabolism of smaller carbohydrate molecules, modification and degradation), synthesis of fatty acids, lipids, secondary metabolism (lignin and terpenes), nucleotides, tricarboxylic acid cycle (TCA), sugars (starch), metabolism of reactive oxygen species (ROS), nitrate and iron contents. Additionally, several metabolic processes involving downregulated DEGs were identified associated with secondary metabolism (lignin), lipids, iron, nucleotides, TCA, metabolism of tetrapyrrolic molecules, fermentation, ROS metabolism and C4/CAM metabolism.

**Figure 4:**
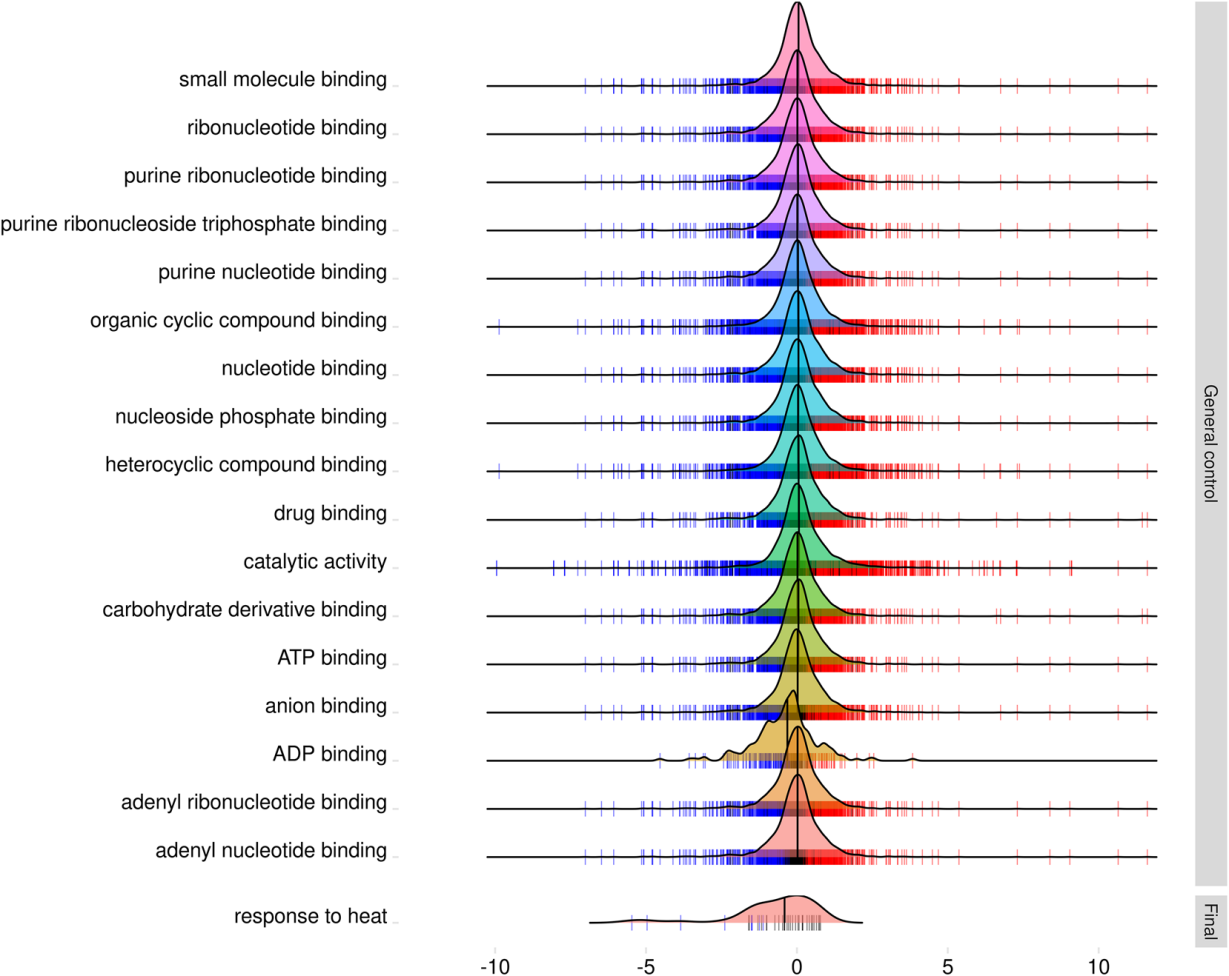
Gene ontology terms enriched in the contrasts “absolute control” and “final time-period”. There was no statistically significant enriched term for the “initial time” contrast (*p* < *0.01*). Density plots depict the distribution of logFC of all the genes for each category. Blue marks are downregulated, red upregulated, and black non-significantly differentially expressed genes.

Based on the enrichment annotation at the three different biological moments of disease progression, the most represented GO terms among the 10,307 DEGs for the “Control” condition are related to metabolic cellular processes such as redox activity (*GO: 0016491*), *purine NTP binding* (*GO: 0035639*), *adenosine 5'-diphosphate binding* (*GO: 0043531*), *coenzyme binding* (*GO: 0050662*) and *drug binding* (*GO:0008144*). There were no enriched GO terms for the “Initial Time-period” condition. However, for the “Final Time-period” among the 366 DEGs, the most enriched GO terms were those related to *serine-like protease activity* (*GO: 0008236*), *response to heat shock* (*GO: 0009408*) and *response to hydrogen peroxide* (*GO: 0042542*) (Fig. 4).

**Figure 5:**
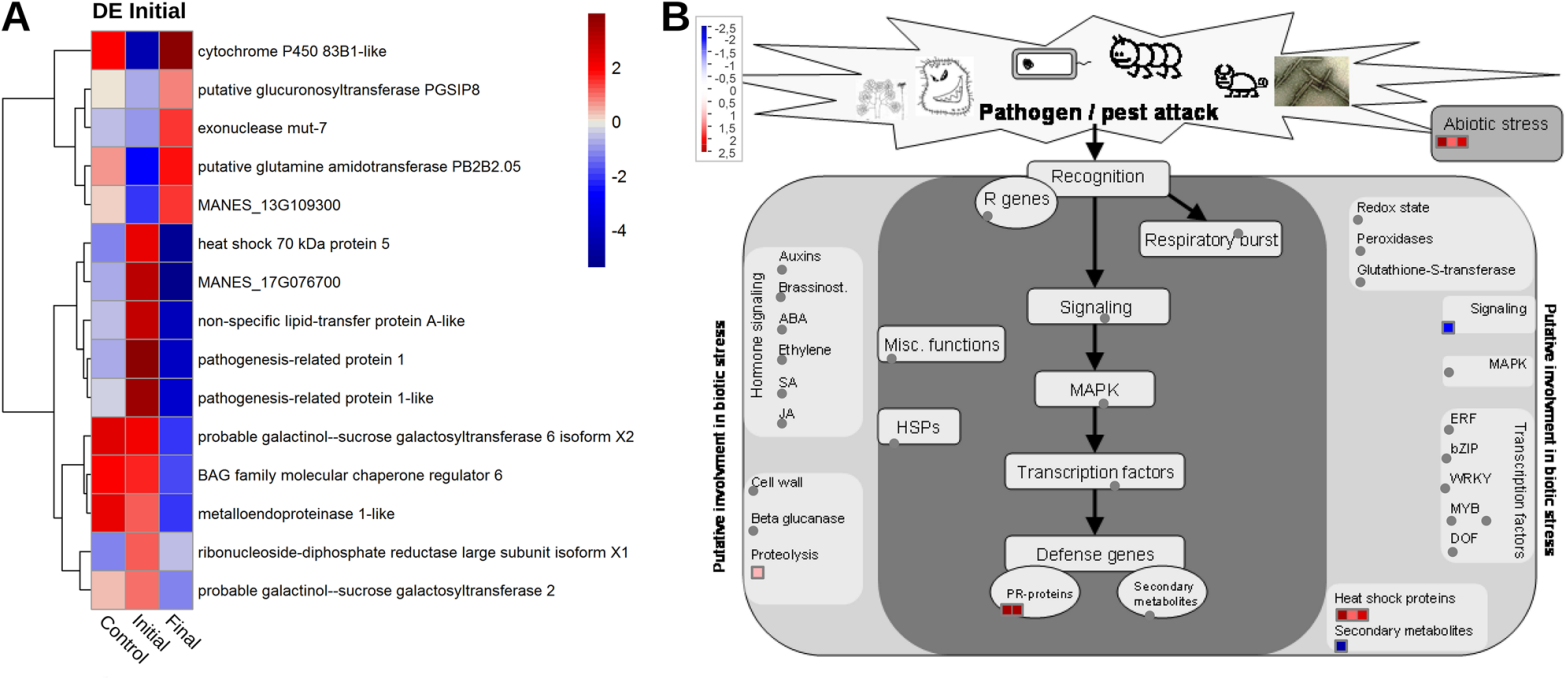
(**A**) Heatmap for differentially expressed genes (DE) at the “Initial Time-period” of the interaction as a function of the LogFC values (*FoldChange*), compared to their expression profile in the samples from the “Control” and “Final – time-period” samples. The blue and red colors represent negatively and positively regulated DEGs, respectively. (**B**) Expression profiles of genes mapped in the biotic stress pathway based on logFC (*FoldChange*). Each square represents a different gene mapped to the same process. Blue dots represent the down-regulated differentially expressed (DE) genes, while the red dots represent the DEGs with up regulation and in gray the genes with experimental indication of involvement in the metabolic pathway.

Based on the comparison of the gene expression of the 15 DEs in the “Initial Time-period” condition with the others, exclusive overexpression and upregulation of genes related to plant defense were identified, such as two proteins related to pathogenesis (PR) and one to heat shock, at the initial time-period, 5–20 DAI, of the interaction, in infested soil (Fig. 5A). Genes expressed at the same basal levels were also found, such as “*AtBAG6* (*BAG family molecular chaperone regulator 6*)” and “*probable galactinol-sucrose galactosyltransferase 6 isoform X2*”, related to programmed cell death and carbohydrate metabolism, respectively. Among these, 15 'Initial Time-period' DEGs, 11 were mapped to metabolic pathways associated with biotic stress (Fig. 5). Among the genes for the initial response to infection, the PR (Pathogen Related) proteins, the heat shock protein and a gene related to proteolysis, are included.

**Figure 6:**
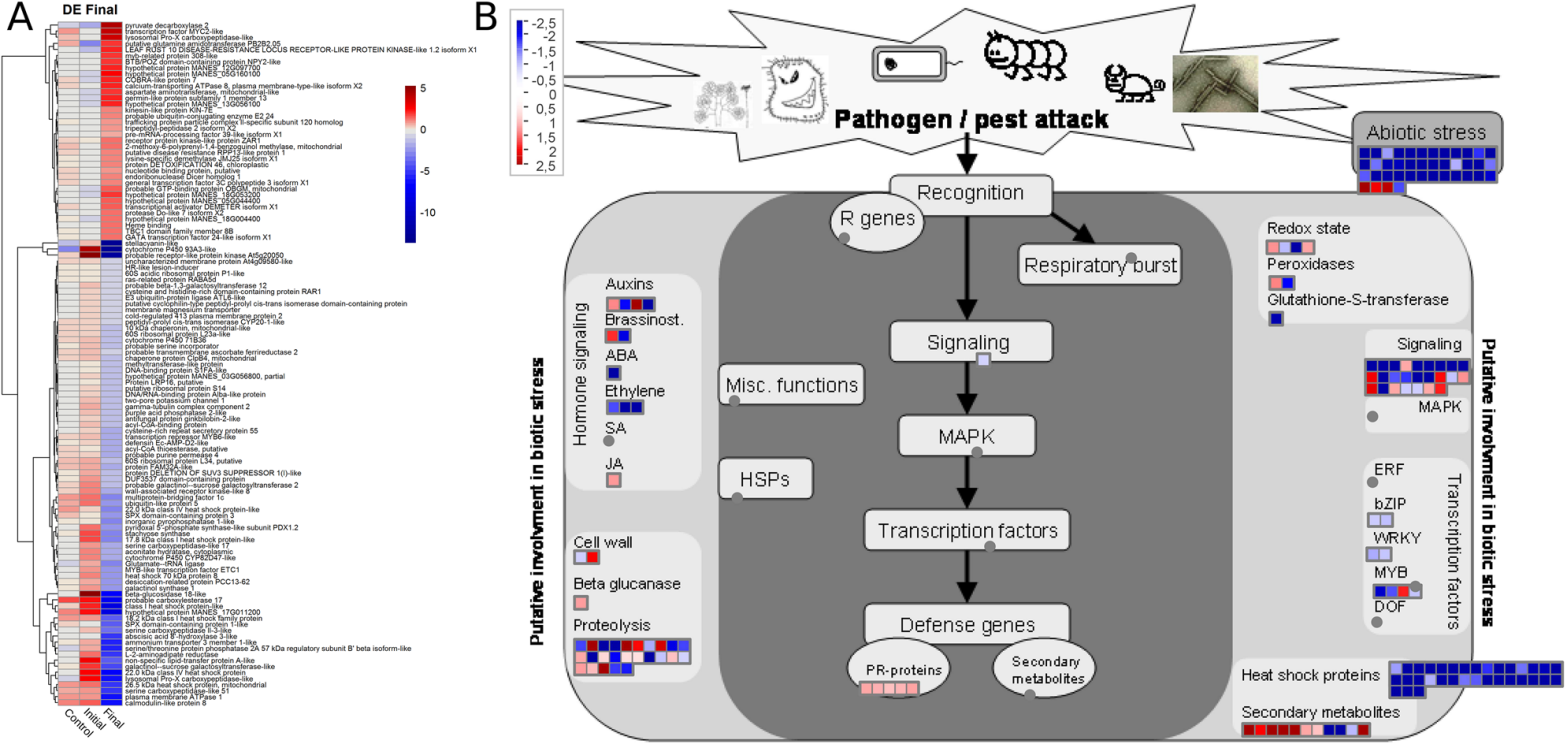
(**A**) Heatmap of differentially expressed genes (DEGs) in the “Final Time-period” of the interaction as a function of LogFC values (*FoldChange*) compared to their expression profile in the “Control” and “Initial Time-period” samples. The blue and red colors represent down-regulated and up-regulated DEGs, respectively. (**B**) Expression profiles of genes mapped in the biotic stress pathway based on logFC (*FoldChange*) in the “Final Time-period”. Each square represents a different gene mapped to the same process. Blue dots represent the differentially expressed genes (DEGs) with down-regulation, while the red dots represent the DEGs with up-regulation and in gray the genes with experimental indication of involvement in the metabolic pathway.

Regarding the differential patterns of gene expression of 366 DEGs in the “Final Time-period” condition, 131 were upregulated (Fig. 6A). From that total, 169 genes were mapped in the biotic stress pathway (Fig. 6B). However, only 42 DEGs were activated and involved in the oxidative stress and peroxidase pathways, in the auxin hormones, jasmonic acid (JA) and brassinosteroids (BR) pathways, defense genes related to secondary metabolites and PR proteins; cell wall reinforcement, beta-glucanase activities and proteolysis, signaling and transcription factors (MYB proteins).

### Candidate genes

**Figure 7:**
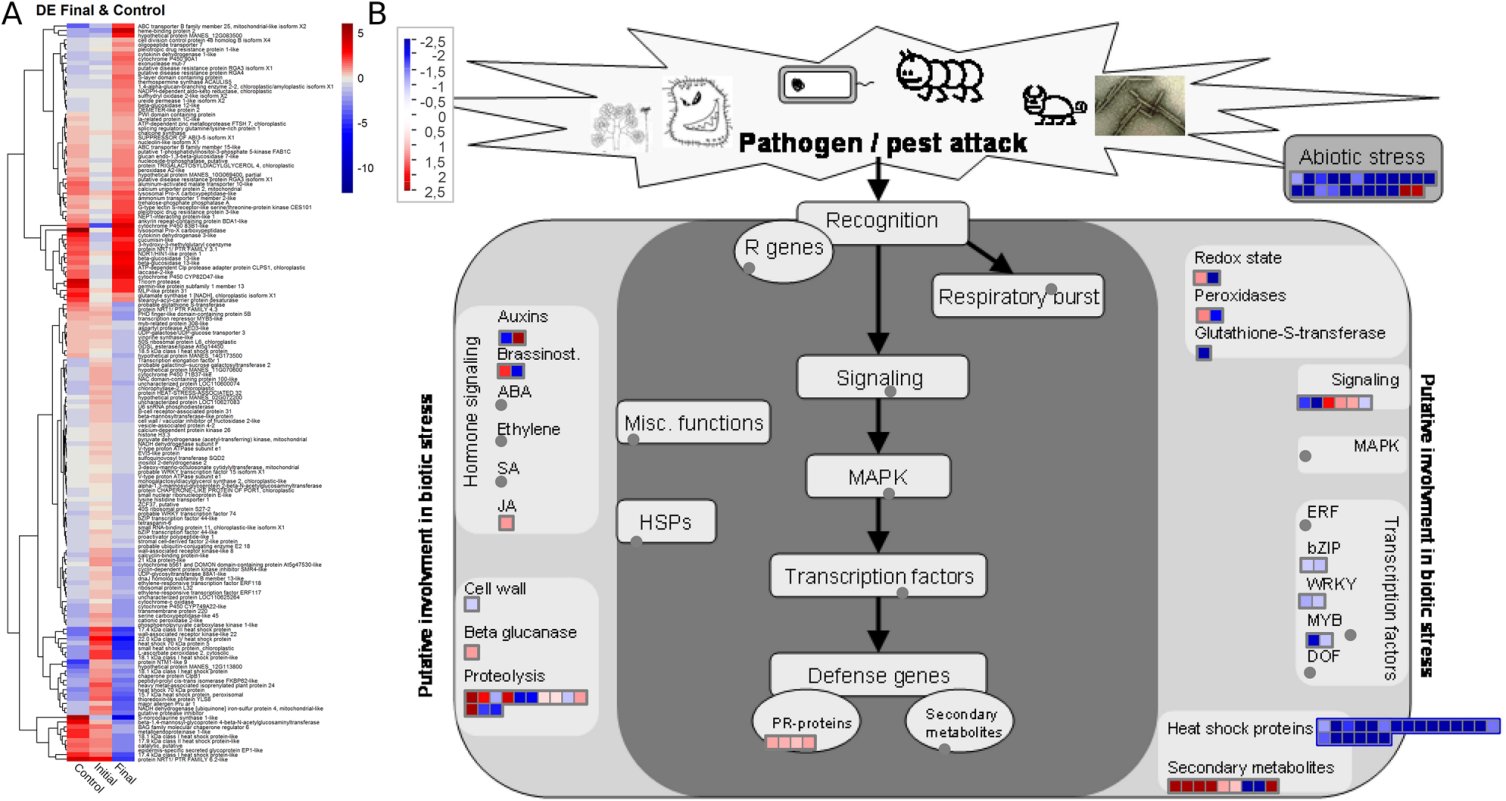
(**A**) Heatmap of differentially expressed genes (DEGs) co-expressed in absolute control and in the initial and final time-periods of the plant/pathogen interaction as a function of LogFC values (*FoldChange*). The blue and red color represents negatively and positively regulated DEGs, respectively. (**B**) Expression profiles of genes co-expressed in the absolute control and in the initial and final time-periods of the plant/pathogen interaction mapped to processes of the biotic stress pathway based on logFC (*FoldChange*). Each square represents a different gene mapped to the same process. Blue dots represent the down-regulated DEGs, while the red dots represent the up-regulated DEGs and in gray the genes with experimental indication of involvement in the metabolic pathway.

Quantitative analysis of transcripts over the time-periods of root rot evolution provided evidence regarding changes in gene expression of the contrasting genotypes, as well as possible candidate genes induced in the resistant genotype. A total of 210 DEGs were identified (Fig. 7A), co-expressed in the “Control” and “inoculated plants (initial and final time-periods of interaction)” treatments. Of these, 85 DEGs were up-regulated, although 22 were considered ‘no hits’ because they did not have an identified protein function in the Genbank data. However, the group of infection response genes includes proteins related to pathogenesis (PR), genes involved in oxidative stress, in the pathways of jasmonic acid, auxin and brassinosteroids (BR) hormones, proteolysis and glucanases, in addition to genes related to transduction of signals and secondary metabolism (Fig. 7B).

The second step of selection was based on events related to responses to biotic stress that were upregulated at baseline levels and maintained overexpression at initial and final time-periods, in addition to those whose expression was restored to baseline levels at ‘final time periods’ of the infection process. Thus, a screening was performed within 63 *up-regulated* DEGs with the identified protein functions, with selection of those with functions related to root elongation, defense and/or resistance to pathogens, cell death, plant immunity and responses to injuries/wounds. Based on these criteria, 18 candidate genes were selected as candidates for further validation via real-time PCR technique (Table 2).

**Table 2:** Candidate genes with potential for validation of gene expression in the *Manihot esculenta* × root rot interaction.

Gene	Gene Ontology -GO	General function
MANES_01G170600	Laccase-2-like	Root elongation under dehydration conditions
MANES_01G250800	BAG family molecular chaperone regulator 6 (AtBAG6)	Basal plant resistance, with programmed responses to cell death and stress
MANES_02G109100	La-related protein 1C-like (LARP1c)	Senescence-associated genes (SAGs) and defense-related genes
MANES_03G129300	ATL27 (NEP1-interacting protein-like 1)	Involved in the early stages of the plant defense signaling pathway
MANES_07G008700	Putative disease resistance protein RGA3 isoform X1	Disease resistance protein
MANES_08G043700	Peroxidase A2-like	Response to pathogen attack, injury and oxidative stress
MANES_08G130900	Germin-like protein (GLP) subfamily 1 member 13	Plays a role in plant defense
MANES_09G064600	Ankyrin repeat-containing protein BDA1-like	Regulation of plant defense responses
MANES_09G146400	G-type lectin S-receptor-like serine/threonine-protein kinase CES101	Fungi response
MANES_10G106500	Protein NRT1/ PTR FAMILY 4.3	Response to nematodes
MANES_11G005800	Aspartyl protease AED3-like	Salicylic acid-mediated response to a pathogen
MANES_11G121200	S-norcoclaurine synthase 1-like	Result of a stimulus by molecules of fungal origin
MANES_11G165400	NDR1/HIN1-like protein 1	Plays a role in plant immunity
MANES_12G144000	Cytochrome P450 83B1-like	Activation of glucosinolate in response to pathogens
MANES_13G135900	Calcium uniporter protein 2, mitochondrial	Activation of cell death pathways
MANES_14G131900	Protein NRT1/ PTR FAMILY 6.2-like	Injury response
MANES_15G004100	Pleiotropic drug resistance protein 3-like	General defense protein
MANES_15G130600	MLP-like protein 31	Response to the presence of a foreign body or the occurrence of an injury

### qRT-PCR analysis

From the selection of candidate genes, a set of 18 primers (Table 3) was designed for gene expression analysis through qRT-PCR considering the treatments (Infested/Healthy) and the post-inoculation interaction time with the pathogens. Among the 18 investigated genes, eight were validated by qPCR: MANES_01G170600, MANES_01G250800, MANES_08G043700, MANES_09G064600, MANES_11G005800, MANES_11G165400, MANES_15G130600 and MANES_15G004100. The analysis revealed distinct expression profiles highlighting the differential response and temporal regulation.

**Table 3:** Primers used in gene expression analysis of the interaction between cassava genotypes and pathogens associated with root rot at different times.

GENE ID	Forward primer sequence (5´- 3´)	GC%	Reverse primer sequence (5´- 3´)	GC%	Amplicon length (pb)
MANES_01G170600	TTGCAGACAGGAGCTGCCCC	65%	CCCTGGCTTCACCTTCAGCC	65%	114
MANES_01G250800	CGCAGCAGGGGAACCAAATG	60%	GCGGAAATAGGGGTAGCACG	60%	164
MANES_02G109100	TAGCTCACCTCGGCATCCTG	60%	AATTTGTGTCCACGGCGAGG	55%	118
MANES_03G129300	GCATTAGGAGGGGCTGCAGT	60%	CTCGCCATCAGCAGCTGATT	55%	147
MANES_07G008700	AGATCGGATTGTGGTGGGGC	60%	CGCCTTAGAGCTTCAGTGGC	60%	199
MANES_08G043700	GGCAGCACTACTACTGGTGC	60%	CGGGGATCGGATTGAAGGGC	65%	126
MANES_08G130900	CTCTGCCTTTGACCCTAGCC	60%	GGTTCTTGCAGAACTTCCCG	55%	95
MANES_09G064600	CGGCGGACCATATACGGAGC	65%	CGCCACCCAGTCAAGAGCCT	65%	117
MANES_09G146400	GGGCAGATGCTGCGGGATTG	65%	TCTACTCGGTTGGCCACCCA	60%	110
MANES_10G106500	AGAGGGAGACCCTCCAATCC	60%	CCACCAAGGAGGGCTAAGAT	55%	212
MANES_11G005800	GCGAGTTGGGAACGCGACGG	70%	CATGGCCAGGCATGTCGTGC	65%	169
MANES_11G121200	TGTTGAGAGTGTGCAGGCGC	60%	GCTCAGCCAGCTCATCACCA	60%	176
MANES_11G165400	ATCTTGCTGCTGCTCTGGGC	60%	CCCCGTCGACAATGCCATGG	65%	177
MANES_12G144000	TTCCCACCAGGTCCTCGAGC	65%	GGTTCAAAGCCCATGCGCAA	55%	134
MANES_13G135900	CTCCCCGGAGCTCCGTTCGA	70%	CCGGTTCCCTCTCCGGCCTA	70%	149
MANES_14G131900	GCACCGGCCTCTTCCTGACA	65%	GCTAACCATCCCTGGCCACC	65%	109
MANES_15G004100	GGCAGCTCAGCTAGTAGGCC	65%	CGCCTCCTTCGGATTGACGG	65%	137
MANES_15G130600	GCGAATGGGGGAAAGAAGGC	60%	GCACCAAGCTTCCATCCCCC	65%	204

**Figure 8:**
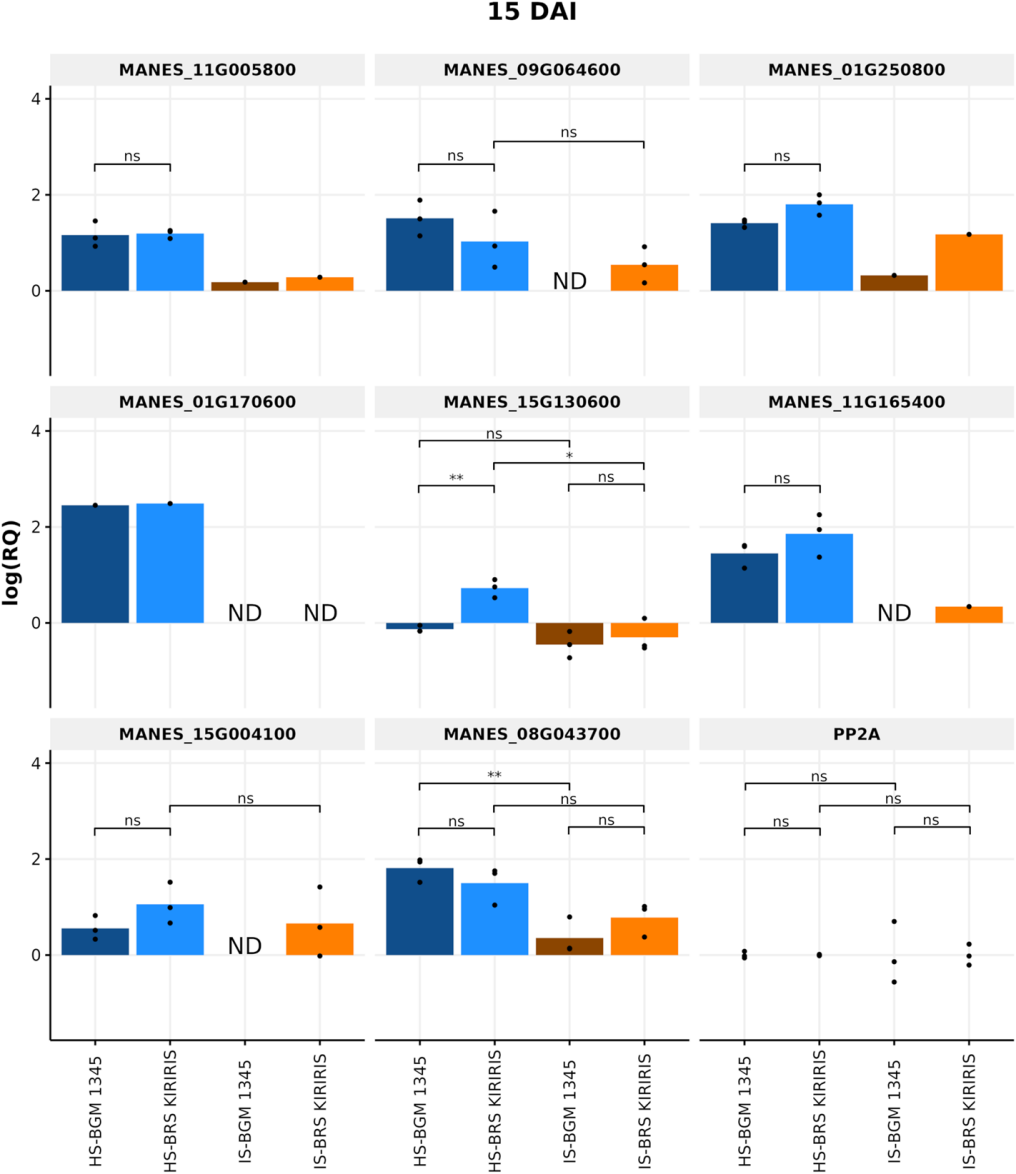
Relative expression levels of eight defense-related candidate genes in cassava contrasting genotypes at 15 days after inoculation (DAI) of pathogens associated with root rot. Averages compared by Student's t test (p < 0.05). ND: "Not detected". IS: Infested Soil. HS: Healthy Soil. The 'protein phosphatase 2A' (PP2A) was used as a reference gene.

bvAt 15 days after inoculation (DAI), MANES_15G130600 (MLP-like protein 31), GO: associated with recovery from infection caused by pathogen infection, showed a significant difference in expression patterns among the studied genotypes (Fig. 8). The resistant genotype (BRS Kiriris) exhibited a higher basal expression of the target gene under the non-inoculated condition compared to the susceptible genotype (BGM-1345). However, considering the inoculated condition, no statistical difference was observed between the expression values of the two genotypes. Furthermore, a reduction in expression values of the resistant genotype was observed in this condition, statistically differing from the non-inoculated treatment. These results suggest that although pathogen inoculation impacted expression patterns similarly in susceptible and resistant genotypes, the resistant genotype demonstrated a differential response, with significantly higher expression in the non-inoculated condition. This may indicate prior preparation or activation of defense mechanisms in the resistant genotype, conferring it an initial advantage in the infection response.

The gene MANES_08G043700 (peroxidase A2-like), associated with GO: response to wounds and pathogen infection, also exhibited distinct expression patterns in response to treatments. Although no statistically significant differences were observed between the two genotypes in both non-inoculated and inoculated conditions, there was a statistical difference in the expression of the target gene by the susceptible genotype between the two conditions. BGM-1345 showed a significant reduction in the inoculated treatment compared to the non-inoculated treatment. This result suggests that the presence of the pathogen negatively impacted the expression of the MANES_08G043700 gene in the susceptible genotype. The reduction in gene expression in the inoculated condition may indicate suppression or interference in plant defense mechanisms in response to pathogen infection, resulting in susceptibility.

Another relevant data is that there was no expression in the inoculated condition compared to the reference gene PP2A for the susceptible genotype for the MANES_09G064600 (ankyrin repeat-containing protein BDA1-like) gene associated with GO: plant defense; MANES_01G170600 (laccase-2-like), associated with GO: lignin biosynthesis; MANES_11G165400 (NDR1/HIN1-like protein 1), GO: plant immunity; MANES_15G004100 (pleiotropic drug resistance protein 3-like), GO: defense protein, making it impossible to perform statistical tests, although it was expressed in the non-inoculated condition. For the resistant genotype, no expression was detected in the inoculated condition, only for MANES_01G170600. These results suggest a possible variation in defense mechanisms between susceptible and resistant genotypes, as well as suppression of defense mechanisms in the susceptible genotype in response to pathogen infection.

**Figure 9:**
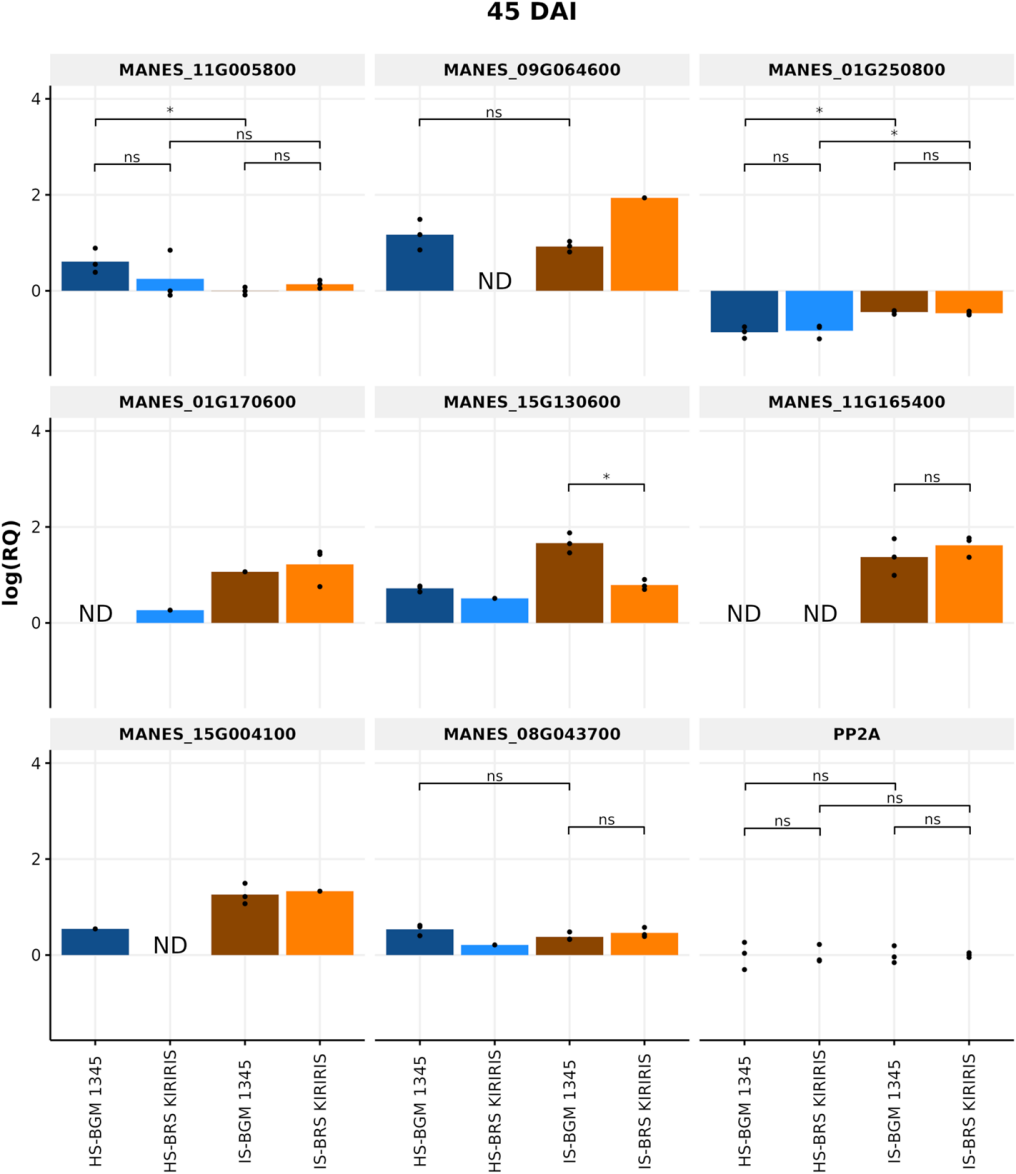
Relative expression levels of eight defense-related candidate genes in cassava contrasting genotypes at 45 days after inoculation (DAI) of pathogens associated with root rot. Averages compared by Student's t test (p < 0.05). ND: "Not detected". IS: Infested Soil. HS: Healthy Soil. The 'protein phosphatase 2A' (PP2A) was used as a reference gene.

Upon evaluating the interaction at 45 DAI (Fig. 9), a statistical difference in the expression of MANES_15G130600 (MLP-like protein 31), was observed. Unlike the interaction at 15 DAI, at 45 DAI, BGM-1345 it showed higher expression values, statistically differing from BRS Kiriris in the inoculated condition. This difference may indicate a compensatory response of the susceptible genotype; however, considering the phenotype observed in the field and under controlled environment experiments, the late expression may not significantly contribute to resistance, highlighting the importance of temporal analysis in understanding the molecular mechanisms underlying cassava resistance.

In contrast, although no statistically significant differences were observed between the two genotypes for the expression of the MANES_11G005800 (aspartyl protease AED3-like) gene in both conditions, there was a statistical difference in the expression of the target gene by the susceptible genotype between the two treatments. BGM-1345 showed a significant reduction in gene expression in the inoculated treatment compared to the non-inoculated treatment, indicating that the presence of the pathogen negatively impacted the expression of the target gene.

In addition to these results, it is important to highlight that the data observed at 45 days after inoculation (DAI) reveal an absence of expression, compared to the reference gene PP2A, of some genes important for plant resistance both in the susceptible and resistant genotypes in the non-inoculated condition. Specifically, for the susceptible genotype, the MANES_01G170600 (laccase-2-like) and MANES_11G165400 (NDR1/HIN1-like protein 1) genes showed no detectable expression. Similarly, for the resistant genotype, the MANES_09G064600 (ankyrin repeat-containing protein BDA1-like), the MANES_11G165400 (NDR1/HIN1-like protein 1), and the MANES_15G004100 (pleiotropic drug resistance protein 3-like) genes, were also not detected, making it impossible to perform statistical analyses. However, it is important to note that both genotypes expressed these genes under inoculated condition.

## Discussion

The understanding of the genetic mechanisms responsible for the different levels of resistance to root rot presented by cassava genotypes is extremely important to assist the characterization of the disease in search for control strategies and selection of promising accessions for the genetic improvement program of the species. Thus, phenotypic data on survival and development of the root system (in direct contact with the soil) in infested areas is the main indicator of resistance of a genotype to root rot. Although field evaluations have some bottlenecks, especially regarding the uneven distribution of inoculum, the data collected here are indicators that the infection process under the same conditions is very different between cassava genotypes considered resistant and susceptible to rot root.

Symptoms in the shoot correlated with the presence of fungal structures in the root during the interaction, which indicates that the variety does not have complete resistance to the disease (immunity profile). Moreover, the infection by root rot pathogen did not prevent the development and expansion of new roots and what is noticed in the susceptible genotype, is the recovery of the diseased plant and the root system, which is constantly in progress. On the other hand, the reduction in the growth of the root system of BGM-1345 at 15 days after planting in infested soil coincided with the detection of fungal presence in its roots. The susceptible genotype (BGM-1345) was not able to emit new roots after infection culminating in the reduction of the root system and even the death of the plant, characterizing extreme susceptibility.

Although the resistance of the BRS Kiriris variety results in a reduction in the symptoms of root rot, its absence of complete resistance reinforces the complexity of resistance to cassava root rot disease with the involvement of many genes, being characteristically a quantitative type of resistance^17^. Comparative analysis of the transcriptome between the two contrasting genotypes over time of interaction provided evidence regarding changes in genes expressed between the resistant genotype and the susceptible genotype, their inferred metabolic functions, as well as possible constitutive candidate genes related to resistance to root rot.

The analysis resulted in a total of 23,912 DEGs with the highest number of DEGs being observed in the 'absolute control' (Time 0-non-inoculated seedlings in non-inoculated substrate), that is, 10,307 DEGs.

The sequencing generated high quality reads with filtering of low-quality reads by Phred analysis. Even with this high quality of reads, the alignment for the BGM-1345 genotype was lower than expected. Our hypothesis is that, since BGM-1345 is a local landrace, with poor agronomic characteristics, highly heterozygotic and susceptible to root rot disease, while BRS Kiriris is an improved variety, the landrace genome might have suffered a disadvantage in comparison to BRS Kiriris since most reference genomes of cassavas favors improved varieties.

Based on functional enrichment analyses, these genes were significantly associated with cellular metabolic processes, such as *oxidoreductase activity* (*GO:0016491*) involved in defense signaling pathways^29^; *NTP binding of purine* (*GO:0035639*), precursors of metabolite biosynthesis, essential in RNA synthesis and energetic coupling reactions^30^; *binding of adenosine 5'-diphosphate -ADP* (*GO:0043531*). Furthermore, associated with components of the structure of leucine-rich repeat receptors (NLRs) involved in plant immune responses^31^, *coenzyme binding* (*GO:0050662*), present in the response of other transcriptomes to biotic and abiotic stresses^32,33^ and *drug binding* (*GO:0008144*), also associated with responses to biotic and abiotic stresses^34^. Therefore, constitutively, enriched GO terms related to signaling pathways and responses to biotic and abiotic stresses were reported in the resistant genotype. This can allow for an early perception of pathogens by the plant with consequent resistance that can allow growth and development, including achieving satisfactory root productivity even when cultivated in areas infested by pathogens that cause root rot.

In contrast, the temporal post-infection responses showed that the number of DEGs in the initial phase of interaction (15 DEGs) was much lower than in the final phase (366 DEGs). In the interaction of cassava × root rot pathogens, the initial response to infection was not related to significantly enriched GOs terms, although transcripts related to heat shock proteins (HSPs) and pathogenesis-related proteins (PR) were positively regulated. HSPs, were also described in the response of cassava genotypes to infection by viruses and bacteria^24,35^. These are biomolecules responsive to various biotic and abiotic stresses responsible for modifying physiological processes and biochemical factors which lead to the development of stress tolerance^36^ as well as pathogenesis-related proteins (PR); major components of the plant's innate immune system, especially of systemic acquired resistance (SAR). PR proteins protect plants from future infections, not only accumulating locally in infected and surrounding tissues, but also in remote, uninfected tissues^37^.

In addition to the mapping of DEGs in the biotic stress pathway, the analysis of the plant's general metabolism indicated the overexpression of genes involved in carbohydrate metabolism and nucleotide synthesis. This overexpression associated with the lack of DEGs involved in the other metabolic processes, may be a strategy of the plant to maintain the minimum energy necessary for its development and ensure that the nutritional needs of the pathogen are not met in the initial phase of the interaction (5 to 20 DAI)^38^. However, at the final time-period (30 to 60 DAI) enrichment of GO terms related to serine-type peptidase activity (GO:0008236) were identified involved in several physiological processes in plants such as hypersensitivity, transduction and differentiation response signal, senescence, degradation and protein processing^39,40^. GOs also related to the response to heat stimulus (GO: 0009408) referring to the increased expression of heat shock proteins involved in the response to biotic and abiotic stresses^36^ and the increase in genes related to the stimulus of hydrogen peroxide (GO: 0042542) involved in cell wall defenses, in hypersensitivity, in the signaling of acquired systemic resistance and in the expression of defense genes^41^ were also found.

On the other hand, the overexpression of genes related to starch biosynthesis, the metabolism of smaller carbohydrate molecules and the TCA cycle, indicates an increase in energy supply at the end of the interaction with the pathogens that cause root rot^42^. Metabolism analysis also identified processes involved in lignin biosynthesis and cell wall modification, which may correspond to plant development and ageing over time, but act as a physical barrier in defense against pathogens^43^.

Regarding the biotic stress pathway, there was an increase in the expression of genes associated with the oxidative process (ROS) and peroxidase; hormonal signaling through the synthesis of auxins, jasmonic acid (JA) and brassinosteroids (BR); defense genes related to secondary metabolites and PR proteins; cell wall reinforcements, beta-glucanase activities and proteolysis, signaling and transcription factors (MYB proteins). An indication that there was progress in the plant × pathogen interaction, is the potential induction of the hypersensitivity response. Triggered by the recognition of biotic elicitors, the hypersensitivity reaction causes death of cells at the site of penetration of the microorganism in addition to the death of some adjacent cells, thus limiting the colonization and advancement by the pathogen^44,45^.

In the resistance mechanism mediated by the hypersensitivity response, the accumulation of reactive oxygen species (ROS), signaling pathways, hormone biosynthesis (especially salicylic acid, jasmonic acid or ethylene), reprogramming of gene expression, especially induction of defense-related genes, such as the production of pathogenesis-related proteins (PR proteins), and genes referring to the biosynthesis pathways of nitrogen-containing compounds, as well as terpenoids, phenols and flavonoids that may result in cell wall thickening and lignification^46,47^ were identified.

These mechanisms were also noted in the biotic stress pathways of the resistant genotype from 30 days after planting in soil infested by black and dry root rot pathogens. Therefore, based on the temporal analysis of the transcriptome of the BRS Kiriris variety, the defense against root rot is possibly composed of a constitutive response present in this variety which possibly allows the rooting and growth of plants in infested areas with penalties to root productivity that are acceptable given the possibility of 100% loss with the use of susceptible varieties. This defense mechanism also seems to be associated with the induced response mainly noted as of 30 days after planting in infested soil.

The transcriptome of infection caused by root rot pathogens reinforces the hypothesis of quantitative inheritance of cassava rot root resistance^17^. From a genotypic point of view, quantitative resistance is based on the combined effect of several genes, each contributing quantitatively to the resistance level of the plant, being able to be influenced by the environment and each other^48^. In general, the perception of the pathogen by the resistant genotype (BRS Kiriris) involved an elaborate system of physical and biochemical mechanisms through the expression of resistance genes followed by the induction of the hypersensitivity response, production of reactive oxygen species, ubiquitination, cell wall reinforcement and hormonal signaling which are fundamental mechanisms during plant immunity^46,47^.

Thus, the selection of candidate genes was based on those DEGs constituting the resistant genotype, positively co-expressed in the initial and/or final moments of the interaction, with functions related to root elongation, defense and/or resistance to pathogens, cellular death, plant immunity, and lesion/injury responses. Due to the quantitative characteristic of cassava root rot resistance, the effects of these genes probably occur concomitantly, making it difficult to separate and identify the real isolated contribution of each mechanism.

However, among the 18 selected DEGs, three genes were found to be involved in defense mechanisms of a structural nature, including cellular and histological defense reactions to limit infection, such as cell wall reinforcement through increased lignification (MANES_01G170600 and MANES_08G043700) adhesion with the plasma membrane (MANES_09G146400), and processes that act as a physical barrier to the penetration of pathogens. MANES_01G170600 (Laccase-2-like) is involved in lignin biosynthesis^49^ through the overexpression of laccases, enzymes expressed in greater numbers in roots compared to other plant organs/tissues. Since the cassava root system is directly affected by black and dry root rot pathogens, possibly the lignin deposit reinforces the cell wall of the roots and, consequently, restricts tissue colonization by pathogens associated with these diseases^43,50^. Likewise, the role of MANES_08G043700 (Peroxidase A2-like), is involved in a wide range of physiological processes such as lignin and suberin formation, auxin metabolism, cell elongation, plant defense, wound healing, as well as the generation of highly reactive oxygen species (ROS) as a plant defense response after pathogen recognition^51,52^. On the other hand, MANES_09G146400 (G-type lectin S-receptor-like serine/threonine-protein kinase) plays a crucial role in mediating cellular response to various environmental stimuli, hormonal signals, and stress perception^53^. Lectin receptor-like kinases contain an extracellular lectin domain that is located in plant cell membranes and play important roles in transmembrane movements, cell wall plasma membrane binding, and responses to pathogens^54,55^.

In addition to genes related to structural nature, 15 related to biochemical responses were selected, such as those involved in mechanisms responsible for the production of reactive oxygen species (ROS), hormonal signaling and production of secondary metabolites, as well as processes that trigger programmed cell death.

Plants have alternative sources of ROS. One of the main responses of plants after the recognition of pathogens and among the selected genes, is the expression of MANES_08G130900 (Germin-like protein subfamily 1 member 13), involved in the response to various biotic and abiotic stress conditions^56^. GLP proteins are present in all organs and developmental stages of plants^57^, and their role in resistance signaling is mainly associated with the production of hydrogen peroxide involved in the oxidative response^58^. Likewise, MANES_13G135900 (*Calcium uniporter protein 2, mitochondrial*), which is associated with mitochondrial calcium homeostasis and plays a key role in cell physiology including regulation of cell bioenergetics, cytoplasmic calcium signals and activation of cell death pathways^59^ triggered by a variety of stimuli including hydrogen peroxide and the production of ROS^60,61^.

Regarding genes involved in hormonal signaling and production of secondary metabolites, MANES_10G106500 (*Protein NRT1/PTR FAMILY 4.3*) and MANES_14G131900 (*Protein NRT1/ PTR FAMILY 6.2-like*) encode the “NRT1/PTR FAMILY” proteins, which were originally identified as nitrate or di/tri-peptide transporters^62^. However, recent studies show that this family also transports the plant hormones auxin, abscisic acid (ABA), gibberellin (GA) and jasmonic acid (JA), as well as secondary metabolites (glucosinolates) involved in a wide range of physiological processes including plant defense against pathogens^63,64^.

The MANES_15G004100 (*Pleiotropic drug resistance protein 3-like*) gene encodes a pleiotropic drug resistance transporter (PDR) found only in fungi and plants^65^. PDR transporters are involved in plant constitutive and induced defenses, probably by transporting antimicrobial secondary metabolites to the cell surface^66,67^. MANES_15G130600 (*MLP-like protein 31*) encodes major latex proteins/ripening-related proteins (MLP/RRP) known to be involved in a wide range of biological processes of plant development and various stress responses^68^. Although they are ubiquitously present in plants, the greatest accumulation of MLP is in the root^69^. Overexpression of these proteins amplify defense signaling, including ethylene, jasmonic acid, and salicylic acid pathways^70,71^. Moreover, MANES_11G005800 (*Aspartyl protease AED3-like*), is associated with systemic acquired resistance (SAR) signaling through salicylic acid (SA)-mediated responses^72^.

The involvement of AED3 aspartyl protease in acquired systemic resistance against fungal and herbivore invasion has been extensively studied^73,74^ and also the gene MANES_11G165400 (*NHL1—NDR1/HIN1-like protein 1*), which plays crucial roles in plant responses to biotic stress^75,76^. The overexpression of genes that are members of the NHL family is related to host resistance by activating hormonal signaling pathways, such as abscisic acid, salicylic acid, jasmonic acid and ethylene^76,77^.

MANES_11G121200 (*S-norcoclaurine synthase 1-like*) encodes norcoclaurine synthase (NCS); a key enzyme in the biosynthesis of benzylisoquinoline alkaloid (BIA), one of the most important secondary metabolites in plants^78^. The NCS is a member of the family of pathogen-related proteins (PR-10)^37^, widely described as a component of the innate immune system in plants, expressed mainly under pathogenic or stress conditions of environmental issues^79,80^.

In addition to the functions described here, hormonal signaling is also involved in processes that trigger programmed cell death. The MANES_02G109100 gene (*la-related protein 1C-like—LARP1c*), for example, is involved in the regulation of premature leaf senescence, in the up-regulation of defense-associated PR genes^81,82^ and salicylic acid biosynthesis, promoting cell death during plant defense^83^. Likewise, MANES_03G129300 (*ATL27*), described as a member of the E3 ubiquitin ligase subfamily^84^ is involved in the initial steps of the plant defense signaling pathway^85,86^. Upregulation of ATLs (Arabidopsis Toxic in Yeast), particularly during plant-pathogen interactions, is related to hormonal signaling^87^ and also to programmed cell death^88^.

Similarly, MANES_01G250800 encodes *AtBAG6* (*BAG family molecular chaperone regulator 6*), a calmodulin-binding protein (CaM) which is proteolytically activated to trigger basal immunity and induce programmed cell death^89,90^. In the infection process, the upregulation of AtBAG6 triggers autophagic cell death and consequently host resistance^91,92^. On the other hand, MANES_09G064600 (*Ankyrin repeat-containing protein BDA1-like*) is a plant immunity regulator, which activates cell death and defense responses constitutively^93^.

Proteins containing ankyrin repeat domain (ANK) are associated with quantitative resistance to different pathogens, including soil-borne pathogens^94,95^, which are the causal agents of cassava black and dry root rots. Also, within the spectrum of mechanisms that act as a toxic barrier to pathogens is MANES_12G144000 (*Cytochrome P450 83B1-like*), a gene necessary for glucosinolate activation in plant response to pathogens^96^. This chemical compound belongs to the class of phytoantecipins which are antimicrobial compounds constitutively produced by the plant without the need for the presence of the microorganism^97,98^. Glucosinolate, when broken down by the enzyme myrosinase, is converted into toxic isothiocyanates (ITCs), which directly protects plants against pathogens and herbivores^99^.

Finally, MANES_07G008700 (*putative disease resistance protein RGA3 isoform X1*), which is an analogue of disease resistance genes (RGA) with conserved domains and structural features that have specific roles in pathogen-host interactions^100^. RGAs specifically target effector proteins from the pathogen within the host cell via the effector-triggered immunity (ETI) response, and thus mediate immunity triggered by pathogen/microbe-associated molecular pattern (PAMP/MAMP) and (PTI/MTI)^101^.

The information provided in this study underscores the complexity of the molecular mechanisms underlying cassava resistance to root rot pathogens. The differential expression patterns observed in response to pathogen inoculation and among genotypes highlight the dynamic nature of plant-pathogen interactions emphasizing the importance of considering both genetic variation and temporal dynamics in these interactions.

Furthermore, the absence of detectable expression for certain genes in the non-inoculated condition, particularly in the susceptible genotype, suggests a potential basal level of defense in the resistant genotype. This phenomenon warrants further investigation to elucidate the underlying regulatory mechanisms and their implications for enhancing resistance breeding strategies in cassava.

Based on their functions, the 18 genes selected here are potentially involved in cassava resistance to root rot, and to our knowledge, this is the first proposed resistance model for the *M. esculenta* × root rot pathosystem. The understanding of these resistance mechanisms, including the biochemical pathways and mainly the identification and validation of these candidate genes will substantially contribute to cassava genetic breeding programs aiming to implement selection strategies assisted by molecular markers for the development and selection of resistant cassava genotypes to root rot disease.

The selection of the genes described here will not only contribute to the use of functional molecular markers associated with resistance, but will also make it possible to screen germplasm and segregating populations in a way that does not depend on inoculation or a more appropriate moment for the overexpression of genes associated with resistance. This will imply in cost and time reduction in the stages of identification of the most promising cassava clones in terms of resistance to root rot.

## Material and methods

### Plant material and experimental site

Aiming to compare the *Maniho*t × root rot disease interaction, two genotypes previously evaluated on field trials and by means of genomic selection were chosen: (i) cultivar BRS Kiriris—code BRA 00073992-0 (https://av.cenargen.embrapa.br/avconsulta/Passaporte/detalhes.do?ida=91789) which is widely planted in Northeastern Region in Brazil as a source of resistance to root-rot with the best ratio between above ground and fresh root yield, meaning that it expresses resistance at field level^26,102^ and (ii) the germplasm accession BGM-1345—code BRA 00077536-1 (https://av.cenargen.embrapa.br/avconsulta/Passaporte/detalhes.do?ida=96632), extremely susceptible to soil-borne diseases^26^.

The experiment was carried out at the Plant Pathology Laboratory and in the Advanced Biology Center (NBA), both located at Embrapa Mandioca e Fruticultura in Cruz das Almas, Bahia, Brazil (12°’0′52″5″ S, 39°’5′22″6″ W, 222 m asl).

All experimental research and field studies involving plants, whether cultivated or wild, were conducted in compliance with applicable institutional, national, and international guidelines and regulations. Permission for the collection of plant material was obtained from SISGEN A8C56D4.

### Plant material preparation for phenotyping

Cassava seedlings from both genotypes were obtained from stem cuttings (5 cm) sprouted and enrooted in 75 mL plastic tubes containing vermiculite and commercial substrate (Vivatto, Technes). The substrate was sterilized by autoclaving at 121 °C for 20 min 48 h previously to the planting of the stem cuttings. The seedlings were grown in an automated thermal chamber for 45 days with 40 °C average temperature, daily variation from 28 to 55 °C, and 60 ± 3% relative humidity.

### Inoculum preparation, inoculation and root clarification

The pathogens were isolated from soil samples gathered from an area naturally infested with root rot-causing pathogens. These samples were collected from a site dedicated to selecting resistant sources over a decade of continuous cassava cultivation in the city of Umbaúba, situated in the state of Sergipe, Brazil (11°22′37.9″ S, 37°40′29.6″ W). The sampling took place at the experimental field of Embrapa Tabuleiros Costeiros during the years 2016 and 2017. Each isolate (six isolates from black rot and 54 from dry rot—Table S2) was cultivated as a single colony in Petri dishes containing PDA medium (200 g L^−1^ of Potato; 20 g L^−1^ of Dextrose and 20 g L^−1^ of Agar), and incubated in growth chamber at a temperature of 25 ± 2 °C with 12 h of light for 7 days. Primary inoculum of each isolate was placed in 500 g sterilized rice grains. In order to prepare the rice medium, the total amount needed of rice grains was previously moistened by adding water directly to the grains (100 mL of water/50 g of rice) and kept for 10 min, followed by discharge of the residual water, and storage in transparent plastic bags using 500 g portions, followed by autoclave sterilization for 20 min at 120 °C. After cooling, 30 mL of spore suspension from each isolate associated with dry and black rot was added to individual rice grains in plastic bags and incubated in growth chamber (BOD) for a seven-day period, at 25 ± 2 °C, under a 12-h photoperiod and 70 ± 10% humidity multiplication. The plastic bags were manually shaken daily to obtain homogeneous colonization of the substrate. For the infestation procedures, the isolates previously grown in rice grains were mixed in a bucket and the same weight of each isolate added.

The plastic bags for the seedlings were filled with 2 kg of naturally infected soil (collected from an area with a history of disease incidence) plus a mixture of 100 g of rice grains infested by isolates associated with black and dry root rot (6 × 10^5^ conidia mL^−1^). As a control treatment, plastic bags were filled with sterilized soil and no infested rice. Soil sterilization was performed by two successive autoclavings at 120 °C with an interval of 24 h between the procedures. After 45 days of planting, the seedlings (all rooted) produced in an automated thermal chamber were transplanted into bags containing infested soil (Treatment 1) and sterilized non-infested soil (Treatment 2). The experiment was kept in the greenhouse and the seedlings irrigated with distilled sterilized water every two days for 60 days.

Samples were collected at eight different time-periods: 0, 5, 10, 15, 20, 30, 45 and 60 days after inoculation (DAI), chosen based on disease dynamics in the field^26^. For each collection time, nine inoculated plants and nine controls were used for the molecular analyses. Plant roots were detached, washed in autoclaved distilled water, packed, identified, frozen in liquid nitrogen and stored in ultrafreezer (− 80 °C).

For visualization of internal colonization of the cassava roots by the pathogens, a clarification procedure was conducted, followed by staining of the fungal structures^103^. Root samples were washed in running water and cut into 2–4 cm long fragments to assess the colonization by fungi isolates associated with root rot disease. For clarification and staining of pathogen structures, three plants from each treatment (inoculated and control) were used. Root fragments were placed in recipients containing KOH solution (10%), heated on a hot plate (Quimis) for one hour (90 °C) for bleaching. Then, the roots were washed in distilled water and acidified in HCl solution (1%) for 4 min at room temperature. Subsequently, the solution was discarded and the roots stained with Trypan Blue solution of 0.05% lactoglycerol (2:1:1; v:v:v; lactic acid:glycerin:water) at room temperature for 24 h. Afterwards, the dye was discarded and the roots immersed in a lactoglycerol solution (2:1:1; v:v:v; lactic acid:glycerin:water) to remove excessive dye. The clarified roots were visualized and photographed under a light microscope (Leica Microsystems model DM500).

### Total RNA extraction

RNA extraction was conducted using the CTAB (2% CTAB; 100 mM Tris–HCl pH 8.0; 50 mM EDTA pH 8.0; 1.4 M NaCl)^104^ protocol with modifications^105^. Total RNA was extracted from the roots of inoculated and non-inoculated cassava plants. 300 mg of tissue was macerated in liquid nitrogen and 1.5 mL of CTAB buffer added to a 2.0 mL microtube and then incubated at room temperature (26 ± 2 °C) for 15 min. Subsequently, the samples were centrifuged at 13.000 rpm for 5 min and 800 µL of the extract was collected and transferred to 2.0 mL Eppendorf microtubes, with subsequent addition of 800 µL of chloroform: isoamyl alcohol (24:1, v:v), vortexed and the mixture centrifuged at 13.000 rpm for 10 min.

The supernatant (600 µL) was transferred to a new microtube (1.5 mL) and 360 µL of isopropanol was added, and the microtubes kept at − 20 °C for 20 min. The samples were then centrifuged at 13.000 rpm for 10 min and the supernatant discarded. The pellet was washed with 70% ethanol (700 µL), kept at − 20 °C for 10 min and centrifuged at 13.000 rpm for 10 min. Ethanol was discarded and the pellet allowed to dry at room temperature. Analysis of the integrity and concentration of total RNA was verified by electrophoresis in a 1% agarose gel in 1 × SB buffer (10 mM NaOH pH 8.5; adjusted with boric acid) at 3 V cm^−1^, applying an aliquot of 2 µL of total RNA from each sample. 700 ng of total RNA from each sample was treated with DNAse I (Amplification grade, Thermo Fisher Scientific, USA). After finding no signs of degradation 1 U μL^−1^ of DNAse, buffer, 1 U of RNAseout (Invitrogen) and sterile ultrapure water (Mili-Q) treated with 0.01% DEPC, were used for a final volume of 20 μL. The reaction was incubated in a Veriti® thermal cycler (Applied Biosystems) at 37 °C for 40 min. Then 2 μL of 25 mM EDTA was added, incubated at 65 °C for 10 min, followed by cooling to 4 °C. Half of the volume of total RNA treated was used for cDNA synthesis and the other half stored in an ultrafreezer (− 80 °C).

### Library construction and RNA sequencing

**Figure 10:**
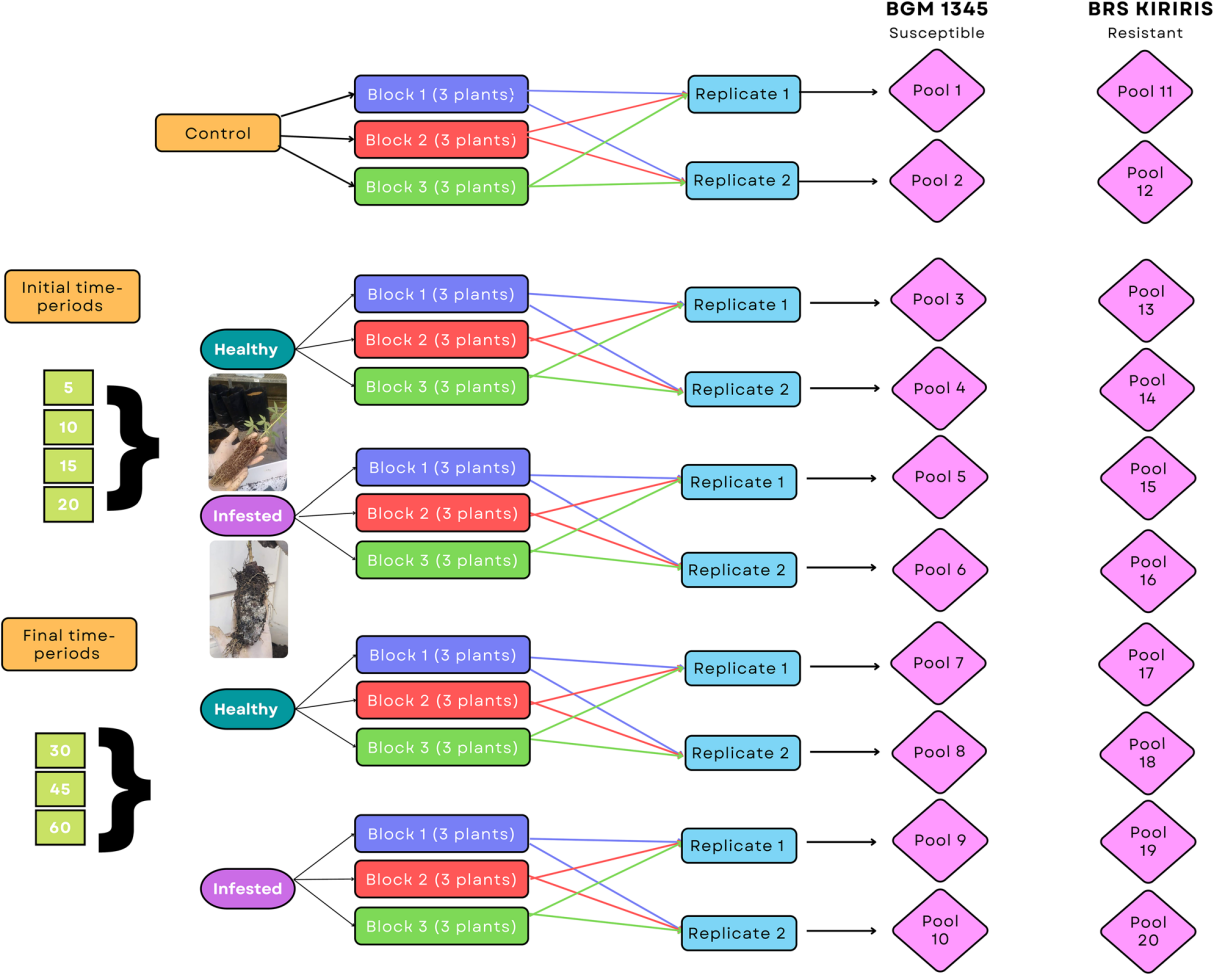
Flow chart depicting the different time-periods and obtainment of pools. Two contrasting varieties for resistance to CRRD, with and without inoculation, were used. The initial time-period was composed by samples collected at 5, 10, 15 and 20 DAI and the final, time-periods at 30, 45 and 60.

The RNA-Seq transcriptional profile analysis of the treatments were derived from an equimolar pool, including the absolute control (time ‘0’), initial time-periods (‘5’, ‘10’, ‘15’ and ‘20’ DAI) and final time-periods (‘30’, ‘45’ and ‘60’ DAI). Afterwards, cDNA libraries were constructed for both genotypes, BRS Kiriris and BGM-1345, produced in infested soil and without infestation (sterilized soil), in the initial and final time-periods, in addition to the absolute control. Each treatment was analyzed in biological duplicates^106–108^, totaling 20 libraries (Fig. 10).

The gene expression profiles of the 20 libraries were obtained based on the RNA-Seq technique, which allowed the quantification and identification of differentially expressed genes (DEGs) potentially related to root rot resistance. The sequences generated by the Cofactor Genomics company (Saint Louis, Missouri, USA) using an Illumina HiSeq xten/NovaSeq 6000 sequencer, were analyzed for quality using FastQC, 1.8 (https://www.bioinformatics.babraham.ac.uk/projects/fastqc/). Raw data generated on the sequencing platform contained a small percentage of low-quality data, which was then filtered to get high-quality data to generate clean reads. The raw data were processed as the following steps by using Pear software (v0.9.6): 1) Filter low quality reads, (more than 15% bases with Phred quality score less than 30); 2) Filter reads with N content greater than 8% reads.

For reference-guided analysis, the sequencing reads were aligned against the cassava reference genome v6.1^28^ deposited in the Phytozome v11.0 database, using STAR software (https://pubmed.ncbi.nlm.nih.gov/23104886/). The count of fragments mapped to each gene was performed using the HTseqe (https://htseq.readthedocs.io/en/master/count.html). Finally, a matrix was set up with the counts of the mapped fragments of each gene in each sample. This table was then used for the analysis of differentially expressed genes (DEGs).

### Differentially expressed gene analysis

After obtaining the count matrix, the analysis of differentially expressed genes was performed using the ‘edgeR’ package implemented in the R 4.0.3 environment^109^. Initially, for each genotype, the samples were classified into five treatments: (C) Control, (HI) Healthy Initial, (II) Infested Initial, (HF) Healthy Final and (IF) Infested Final. Annotation to represent the gene abundances per sample by πg, t, was adopted with the first index to denote a genotype and the second for a specific treatment, e.g., πBRS.HI represents the sample BRS Kiriris with HI treatment. Then, multidimensional scaling (MDS) analysis was performed to infer about the variability of the samples, separating technical and biological samples variances. This variance was based on the difference in expression of the 500 most differentially expressed genes among the analyzed genotypes with distances calculated from the gene expression levels.

From the classification of the samples, the expression levels were adjusted using a generalized linear model—glmLRT (generalized linear model—Likelihood Ratio Test), based on the negative binomial distribution, which models the data dispersion by including different parameters for mean and variance. In order to better exploit resistance-related genes against root rot pathogens, the following comparisons were made between the contrasting treatments: (1) Absolute Control: πBRS.C − πBGM.C; (2) Initial Time: (πBRS.II − πBRS.HI) − (πBGM.II − πBGM.HI); and (3) Final Time: [(πBRS.IF − πBRS.II) − (πBRS.HF − πBRS.HI)] − [(πBGM.IF − πBGM.II) − (πBGM,HF − πBGM.HI)]. Absolute Control compares the basal expression between susceptible and resistant under controlled conditions. Initial Time and Final time-periods tests check for the interaction of infection and genotype at different time points, having as baseline healthy plants also grown in sterile conditions sampled in the same manner and age as infected ones. Thus, the overall purpose of the latter contrasts is to detect differentially expressed genes elicited by infection or disease in a genotypic-specific manner.

For each contrast, the differential expression profile was based on the likelihood ratio test^110^. Finally, the False Discovery Rate—FDR (p-value ≤ 0.05)^111^ was used to correct for multiple testing and to control the occurrence of false positives, improving the accuracy in the identification of most significant genes. The differentially expressed, activated or repressed genes between the compared treatments, were used to generate mean-difference graphs, which relate the log fold-change (log FC) and represent the relative differences between groups, with the mean expression level of the genes measured by the mean logarithm of counts per million (log CPM).

For each one of the three contrasting treatments (1—‘Absolute Control’, 2—‘Initial Time’ and 3—‘Final Time’) a list of differentially expressed genes between the resistant and susceptible variety, was generated. The three gene groups were then compared and illustrated in a Venn diagram using the “VennDiagram” package, in the R 4.0.

### Functional assessment of differentially expressed genes

After identifying the genes with increased (up-regulated) or decreased (down-regulated) expression, the biological and molecular processes modulated in response to the changes in which the genotypes were found (soil conditions and plant/pathogen interaction time), were mapped.

Gene ontology (GO) analysis was performed with the Blast2Go software^112^, as well was hierarchical ontology classification with the MapMan software^113^. For these steps a functional annotation file for the reference genome and the appropriate MERCATOR v.3.6 ontologies^114^, were used; the latter is based on information contained in functional annotation databases, on a genome-wide scale. The comparison of the log fold-change values of the differentially expressed genes (DEGs) in each condition and the molecular function of each DE gene were presented in the form of a heatmap, using the pheatmap package in the R 4.0.3 software^109^.

### Candidate genes

The candidate genes to be validated were selected based on three criteria: (i) DEGs in common between the ‘Control’ and ‘Inoculated’ contrasts (Initial and final time-periods of the interaction); (ii) the Log2 Fold-Change differential expression, selecting only the overexpressed genes (up-regulated), and (iii) the selection of genes with relevant potential for signaling pathways or direct defense responses of plants to pathogens based on a more in-depth search of the functions through the UniProt platform^115^.

### qRT-PCR primer design, conditions and analysis

The primers were designed using the online platform Benchling (https://www.benchling.com/) taking into account the described estimates of suitable melting temperature for qPCR, GC content, ΔG of primer hybridization, and amplified fragment length no longer than 220 bp. The primers were designed based on the target genes selected from our transcriptomic analyses using the available genomic sequence of *M. Esculenta Crantz*. Next, the candidate primers were subjected to a blastn query against the genome to avoid non-specific binding and duplicated genes. The 'protein phosphatase 2A' (PP2A) was used as a reference gene selected from previous studies on gene expression in cassava^116^.

### cDNA synthesis and quality control

For the qRT-PCR analysis, RNA samples from two genotypes (BGM-1345 and BRS Kiriris) were used at both the initial period (15 DAI) and the final period (45 DAI), under both inoculated and non-inoculated conditions. Thus, the analysis comprised 2 (genotypes) × 2 (time points) × 2 (treatments) × 18 (genes) × 3 replicates.

The cDNA synthesis was performed using 10 μL of DNAse-treated RNA supplemented with 1 μL of 50 μM poly-T primer (oligo-dT, 18 bp), 1 μL of 10 mM dNTPs, and 3 μL of sterile Milli-Q water, in a final volume of 15 μL. The mixture was then incubated at 65 °C for 5 min followed by cooling to 4 °C for 5 min. For reverse transcription, 20 U of Ribolock RNase Inhibitor (Fermentas) and 200 U of Revertaid Premium Reverse Transcriptase (Fermentas) supplemented with the appropriate 5X RT Buffer (Fermentas) were added to a final volume of 20 μL. The reaction was incubated in the Applied Biosystems thermocycler (Model Veriti) at 60 °C for 30 min followed by 85 °C for 5 min to deactivate the reaction. After synthesis the cDNAs were stored at − 20 °C.

In order to verify the amplification identity of the primers designed for real-time quantitative PCR (qPCR) gene expression analyses an amplification reaction, was conducted. The reaction mixture contained 3 μL of cDNA at a 1:10 dilution (v/v), 0.2 mM dNTPs, 0.2 μM of each primer, 2 mM MgCl_2_, 1 U of Taq DNA polymerase (Fermentas) in appropriate buffer and sterile Milli-Q water in a final volume of 25 μL. Amplification was carried out using the Applied Biosystems thermocycler (Model Veriti) with the following cycling conditions: initial denaturation at 94 °C for 4 min; 35 cycles of denaturation at 94 °C for 30 s, annealing at 60 °C for 30 s, and extension at 72 °C for 30 s; and a final extension at 72 °C for 5 min. The amplification product was visualized on 1% agarose gel with 1X SB buffer at 3 V cm^−1^.

The gene expression quantification was performed using the ABI PRISM 7500 Real-Time PCR system (Applied Biosystems) with SYBR Green detection. Negative controls consisting of contaminant-free water samples were included in place of cDNA, and melting curves were generated for all analyses. Each reaction included 1.0 μL of cDNA, 0.3 μL of each primer, 5.0 μL of SYBR Green Master Mix with ROX (Invitrogen), and 3.4 μL of water, resulting in a final volume of 10.0 μL per sample. The amplification protocol consisted of an initial cycle of 2 min at 50 °C followed by denaturation for 10 min at 95 °C, then 40 cycles of 15 s at 95 °C and 1 min at 60 °C and finalizing with 15 s at 95 °C with fluorescence signal detection at the end of each extension step. Data were collected and stored using the 7500 Fast Software (Version 2.1).

Melting curve analysis was performed after completion of the reaction cycles with melting temperatures ranging from 72 to 95 °C. For each gene studied, the entire qRT-PCR experiment was conducted using different cDNAs obtained from each sample with three technical replicates for each. Results were normalized using cycle threshold (CT) values obtained from the expression of the reference gene (PP2A) employing the comparative CT method.

The expression values for each sample were normalized using the reference gene and the quantitative expression variation of the target genes in the inoculated samples at each time point was calculated relative to the non-inoculated samples. Normalization was performed using the equation ΔCT = CT (target gene) − CT (endogenous control). Calibration was determined by the formula ΔΔCT = ΔCT (sample) − ΔCT (calibrator) with the calibrator being one of the samples used as the basis for comparative expression results. Relative quantification was obtained using the formula RQ = 2^−ΔΔCT^. Following real-time PCR amplification, each amplification product was analyzed by the dissociation curve to ensure that for each gene and genotype the amplified product did not exhibit nonspecific bands or primer dimer formation.

The RQ estimates were inputted for the case of missing data using the arithmetic mean of the remaining data points per group. We performed a Student T test to assess whether log(RQ) was significantly different between groups with n = 3 samples within the detection threshold. The p-values were further corrected for multiple comparisons.

## Conclusion

Plants of the BRS Kiriris variety considered phenotypically resistant to root rot restricted the progression of the disease, limited the occurrence of symptoms in the leaves and allowed the expansion (increase in volume) of the roots, even when under fungal colonization.

Temporal post-infection responses showed that the number of DEGs in the initial phase of interaction (15 DEGs) was much lower than in the final phase (366 DEGs). In addition, GO terms that significantly enriched in the initial response to infection (five to 20 DAI), were not identified.

The cassava defense/resistance response model to root rot disease is based on the induction of genes that encode the hypersensitivity response (HR), acquired systemic resistance (SAR), induction of pathogen-related proteins (PR-Proteins), reinforcements of the cell wall and signaling compounds, such as auxins, jasmonic acid (JA), brassinosteroids (BR) and hydrogen peroxide.

A total of 18 candidate genes were selected and their validation will allow the use of functional molecular markers associated with resistance for further screening of germplasm and segregating populations to identify the most promising cassava clones with resistance to root rot.

### Supplementary Information

Supplementary Tables.

Supplementary Figure S1.

## Data Availability

All datasets generated for this study are presented in the article, supplementary material, and Figshare (https://doi.org/10.6084/m9.figshare.20493144.v1).

## References

[1] Foundation Agricultural Organization (FAO) (2013). Save and Grow: Cassava. A Guide to Sustainable Production Intensification.

[2] Foundation Agricultural Organization (FAO) (2018). Food Outlook: Biannual Report on Global Food Markets.

[3] McCallum EJ, Anjanappa RB, Gruissem W (2017). Tackling agriculturally relevant diseases in the staple crop cassava (Manihot esculenta). Curr. Opin. Plant Biol..

[4] Akrofi S, Akuoko K, Bour K, Nyarko A (2018). Farmers’ knowledge and perception of the dry cassava root rot disease in Brong Ahafo region of Ghana. Ghana J. Agric. Sci..

[5] Boari AJ, Cunha EM, Quadros AFF, Barreto RW, Fernandes AF (2018). First report of Phytopythium sp. causing storage root rot and foliage blight of cassava in Brazil. Plant Dis..

[6] Brito ACQ (2020). Diversity and pathogenicity of Botryosphaeriaceae species associated with black root rot and stem cutting dry rot in Manihot esculenta in Brazil. Eur. J. Plant Pathol..

[7] Mihajlovic M, Rekanovic E, Hrustic J, Grahovac M, Tanovic B (2017). Methods for management of soilborne plant pathogens. Pestic. Fitomed..

[8] Facelli E, McKay SF, Facelli JM, Scott ES (2018). A soil-borne generalist pathogen regulates complex plant interactions. Plant Soil.

[9] Jamilly AB (2014). Different cover promote sandy soil suppressiveness to root rot disease of cassava caused by Fusarium solani. Afr. J. Microbiol. Res..

[10] Huang, X. *et al.* Toxic organic acids produced in biological soil disinfestation mainly caused the suppression of Fusarium oxysporum f sp cubense. *BioControl* **60**, 113–124 (2015).

[11] Silva, J. M. D, Medeiros, E. V. De, Duda, G. P., Barros, J. A. De & Santos, U. J. Dos. Fames and microbial activities involved in the suppression of cassava root rot by organic matter. *Rev. Caatinga* **30**, 708–717 (2017).

[12] Freitas MA (2019). Stem inoculation with bacterial strains Bacillus amyloliquefaciens (GB03) and Microbacterium imperiale (MAIIF2a) mitigates Fusarium root rot in cassava. Phytoparasitica.

[13] Onyeka TJ, Dixon AGO, Ekpo EJA (2005). Identification of levels of resistance to cassava root rot disease (Botryodiplodia theobromae) in African landraces and improved germplasm using in vitro inoculation method. Euphytica.

[14] Oliveira, S. A. S. de, Hohenfeld, C. S., Santos, V. da S., Haddad, F. & Oliveira, E. J. de. Resistance to Fusarium dry root rot disease in cassava accessions. *Pesqui. Agropecuária Bras.* **48**, 1414–1417 (2013).

[15] Awoyemi SO, Afolabi CG, Popoola AR, Odedina JN, Adigbo SO (2016). Performances of improved cassava ( *Manihot esculenta* Crantz) cultivars against root rot disease and yield in cassava-maize intercropping systems under natural infection. Arch. Phytopathol. Plant Prot..

[16] Boas, S. A. V., Hohenfeld, C. S., de Oliveira, S. A. S., da Silva Santos, V. & de Oliveira, E. J. Sources of resistance to cassava root rot caused by Fusarium spp.: A genotypic approach. *Euphytica* **209**, 237–251 (2016).

[17] Brito AC, Oliveira SAS, Oliveira EJ (2017). Genome-wide association study for resistance to cassava root rot. J. Agric. Sci..

[18] Santiago CH, Santana MP, Cairo Junior LR, de Oliveira SAS, de Oliveira EJ (2018). Methodologies for selecting cassava with resistance to dry and black root rot under controlled conditions. Bragantia.

[19] Singh AK, Singh PK, Arya M, Singh NK, Singh US (2015). Molecular screening of blast resistance genes in rice using SSR markers. Plant Pathol. J..

[20] Li C (2012). Transcriptome profiling of resistant and susceptible Cavendish banana roots following inoculation with Fusarium oxysporum f. sp. cubense tropical race 4. BMC Genom..

[21] Wang Z, Gerstein M, Snyder M (2009). RNA-Seq: a revolutionary tool for transcriptomics. Nat. Rev. Genet..

[22] Prochnik S (2012). The Cassava genome: Current progress, future directions. Trop. Plant Biol..

[23] Utsumi Y (2016). Cassava (Manihot esculenta) transcriptome analysis in response to infection by the fungus Colletotrichum gloeosporioides using an oligonucleotide-DNA microarray. J. Plant Res..

[24] Amuge T (2017). A time series transcriptome analysis of cassava (Manihot esculenta Crantz) varieties challenged with Ugandan cassava brown streak virus. Sci. Rep..

[25] Anjanappa RB (2018). Molecular insights into *Cassava brown streak virus* susceptibility and resistance by profiling of the early host response. Mol. Plant Pathol..

[26] Hohenfeld CS, Passos AR, de Carvalho HWL, de Oliveira SAS, de Oliveira EJ (2022). Genome-wide association study and selection for field resistance to cassava root rot disease and productive traits. PLoS ONE.

[27] Lima AM (2018). Expression profiles of defense genes in cassava storage roots upon exposure to Phytopythium sp., causal agent of soft root rot disease. Physiol. Mol. Plant Pathol..

[28] Bredeson JV (2016). Sequencing wild and cultivated cassava and related species reveals extensive interspecific hybridization and genetic diversity. Nat. Biotechnol..

[29] Gómez-Cano F (2019). Gene co-expression network for Xanthomonas-challenged cassava reveals key regulatory elements of immunity processes. Eur. J. Plant Pathol..

[30] Witte C-P, Herde M (2020). Nucleotide metabolism in plants. Plant Physiol..

[31] Wang J (2019). Ligand-triggered allosteric ADP release primes a plant NLR complex. Science.

[32] Feng H (2019). Responses of Fuji (Malus domestica) and Shandingzi (Malus baccata) apples to Marssonina coronaria infection revealed by comparative transcriptome analysis. Physiol. Mol. Plant Pathol..

[33] Zou C (2020). Transcriptome analysis of sugar beet (Beta vulgaris L) in response to alkaline stress. Plant Mol. Biol..

[34] Kaminaka H (2020). Nanofibrillation is an effective method to produce chitin derivatives for induction of plant responses in soybean. Plants.

[35] Muñoz-Bodnar A (2014). RNAseq analysis of cassava reveals similar plant responses upon infection with pathogenic and non-pathogenic strains of Xanthomonas axonopodis pv. manihotis. Plant Cell Rep..

[36] Haq Ul (2019). Heat shock proteins: Dynamic biomolecules to counter plant biotic and abiotic stresses. Int. J. Mol. Sci..

[37] Jain S, Kumar A (2015). The pathogenesis related class 10 proteins in plant defense against biotic and abiotic stresses. Adv. Plants Agric. Res..

[38] Correr FH (2020). Time-series expression profiling of sugarcane leaves infected with Puccinia kuehnii reveals an ineffective defense system leading to susceptibility. Plant Cell Rep..

[39] Nishimura K, Kato Y, Sakamoto W (2017). Essentials of proteolytic machineries in chloroplasts. Mol. Plant.

[40] Silva-Brandão KL (2017). Transcript expression plasticity as a response to alternative larval host plants in the speciation process of corn and rice strains of Spodoptera frugiperda. BMC Genom..

[41] Li D (2013). Ectopic expression of rice OsBIANK1, encoding an ankyrin repeat-containing protein, in Arabidopsis confers enhanced disease resistance to Botrytis cinerea and Pseudomonas syringae. J. Phytopathol..

[42] Selinski J (2018). Alternative oxidase isoforms are differentially activated by tricarboxylic acid cycle intermediates. Plant Physiol..

[43] Arcuri MLC (2020). Genome-wide identification of multifunctional laccase gene family in Eucalyptus grandis: Potential targets for lignin engineering and stress tolerance. Trees.

[44] Stael S (2015). Plant innate immunity: Sunny side up?. Trends Plant Sci..

[45] TrdÃ L (2015). Perception of pathogenic or beneficial bacteria and their evasion of host immunity: pattern recognition receptors in the frontline. Front. Plant Sci..

[46] An C, Mou Z (2012). Non-Host defense response in a novel Arabidopsis-Xanthomonas citri subsp. citri pathosystem. PLoS ONE.

[47] Cheng Y (2012). Characterization of non-host resistance in broad bean to the wheat stripe rust pathogen. BMC Plant Biol..

[48] Niks RE, Qi X, Marcel TC (2015). Quantitative resistance to biotrophic filamentous plant pathogens: Concepts, misconceptions, and mechanisms. Annu. Rev. Phytopathol..

[49] Wang J (2015). The E3 ligase OsPUB15 interacts with the receptor-like kinase PID2 and regulates plant cell death and innate immunity. BMC Plant Biol..

[50] Abou-Elwafa SF, Amin AE-EAZ, Shehzad T (2019). Genetic mapping and transcriptional profiling of phytoremediation and heavy metals responsive genes in sorghum. Ecotoxicol. Environ. Saf..

[51] Pandey VP, Awasthi M, Singh S, Tiwari S, Dwivedi UN (2017). A comprehensive review on function and application of plant peroxidases. Biochem. Anal. Biochem..

[52] Dragana R, Nikola G, Željko D, Gordana A, Olgica N (2017). Separation of peroxidases from Miscanthus x giganteus, their partial characterisation and application for degradation of dyes. Plant Physiol. Biochem..

[53] Sun X-L (2013). GsSRK, a G-type lectin S-receptor-like serine/threonine protein kinase, is a positive regulator of plant tolerance to salt stress. J. Plant Physiol..

[54] Vaid N, Macovei A, Tuteja N (2013). Knights in action: Lectin receptor-like kinases in plant development and stress responses. Mol. Plant.

[55] Zhao T, Wang J, Zhang B, Hou X (2018). Genome-wide analysis of lectin receptor-like kinases in tomato (Solanum lycopersicum) and its association with the infection of tomato yellow leaf curl virus. Plant Mol. Biol. Rep..

[56] Cevher Keskin B (2019). Quantitative mRNA expression profiles of germin-like and extensin-like proteins under drought stress in Triticum aestivum. Int. J. Life Sci. Biotechnol..

[57] El-Sharkawy I, Mila I, Bouzayen M, Jayasankar S (2010). Regulation of two germin-like protein genes during plum fruit development. J. Exp. Bot..

[58] Zhang XY (2013). Relationship between disease resistance and rice oxalate oxidases in transgenic rice. PLoS ONE.

[59] Teardo E (2017). Physiological characterization of a plant mitochondrial calcium uniporter in vitro and in vivo. Plant Physiol..

[60] Van Aken O, Van Breusegem F (2015). Licensed to kill: Mitochondria, chloroplasts, and cell death. Trends Plant Sci..

[61] Penna E, Espino J, De Stefani D, Rizzuto R (2018). The MCU complex in cell death. Cell Calcium.

[62] Tsay Y (1993). The herbicide sensitivity gene CHL1 of arabidopsis encodes a nitrate-inducible nitrate transporter. Cell.

[63] Chiba Y (2015). Identification of Arabidopsis thaliana NRT1/PTR FAMILY (NPF) proteins capable of transporting plant hormones. J. Plant Res..

[64] Corratgé-Faillie C, Lacombe B (2017). Substrate (un)specificity of Arabidopsis NRT1/PTR FAMILY (NPF) proteins. J. Exp. Bot..

[65] Crouzet J, Trombik T, Fraysse ÅS, Boutry M (2006). Organization and function of the plant pleiotropic drug resistance ABC transporter family. FEBS Lett..

[66] Sasse J (2016). *Petunia hybrida* PDR2 is involved in herbivore defense by controlling steroidal contents in trichomes. Plant. Cell Environ..

[67] Pierman B (2017). Activity of the purified plant ABC transporter NtPDR1 is stimulated by diterpenes and sesquiterpenes involved in constitutive and induced defenses. J. Biol. Chem..

[68] Zhang N (2018). Genome-wide evolutionary characterization and expression analyses of major latex protein (MLP) family genes in Vitis vinifera. Mol. Genet. Genom..

[69] Yang C-L (2015). Cotton major latex protein 28 functions as a positive regulator of the ethylene responsive factor 6 in defense against Verticillium dahliae. Mol. Plant.

[70] He S (2020). Major latex protein MdMLP423 negatively regulates defense against fungal infections in apple. Int. J. Mol. Sci..

[71] Holmquist L (2021). Major latex protein-like encoding genes contribute to Rhizoctonia solani defense responses in sugar beet. Mol. Genet. Genom..

[72] Breitenbach HH (2014). Contrasting roles of the apoplastic aspartyl Protease APOPLASTIC, *ENHANCED DISEASE SUSCEPTIBILITY1* -DEPENDENT1 and LEGUME LECTIN-LIKE PROTEIN1 in Arabidopsis Systemic acquired resistance. Plant Physiol..

[73] López-Galiano MJ (2017). Oxylipin mediated stress response of a miraculin-like protease inhibitor in Hexanoic acid primed eggplant plants infested by Colorado potato beetle. J. Plant Physiol..

[74] Niu L, Liu L, Wang W (2020). Digging for stress-responsive cell wall proteins for developing stress-resistant maize. Front. Plant Sci..

[75] Chen Q (2018). StPOTHR1, a NDR1/HIN1-like gene in Solanum tuberosum, enhances resistance against Phytophthora infestans. Biochem. Biophys. Res. Commun..

[76] Liu C (2020). Genome-wide analysis of NDR1/HIN1-like genes in pepper (Capsicum annuum L.) and functional characterization of CaNHL4 under biotic and abiotic stresses. Hortic. Res..

[77] Peng H (2019). Overexpression of a pathogenesis-related gene NbHIN1 confers resistance to Tobacco Mosaic Virus in Nicotiana benthamiana by potentially activating the jasmonic acid signaling pathway. Plant Sci..

[78] Vimolmangkang S (2016). Evolutionary origin of the NCSI gene subfamily encoding norcoclaurine synthase is associated with the biosynthesis of benzylisoquinoline alkaloids in plants. Sci. Rep..

[79] Fernandes H, Michalska K, Sikorski M, Jaskolski M (2013). Structural and functional aspects of PR-10 proteins. FEBS J..

[80] Finkina EI, Melnikova DN, Bogdanov IV, Ovchinnikova TV (2017). Plant Pathogenesis-related proteins PR-10 and PR-14 as components of innate immunity system and ubiquitous allergens. Curr. Med. Chem..

[81] Lim PO, Kim HJ, Gil Nam H (2007). Leaf senescence. Annu. Rev. Plant Biol..

[82] Zhang B, Jia J, Yang M, Yan C, Han Y (2012). Overexpression of a LAM domain containing RNA-binding protein LARP1c induces precocious leaf senescence in Arabidopsis. Mol. Cells.

[83] Na J-K, Kim J-K, Kim D-Y, Assmann SM (2015). Expression of potato RNA-binding proteins StUBA2a/b and StUBA2c induces hypersensitive-like cell death and early leaf senescence in Arabidopsis. J. Exp. Bot..

[84] Serrano M, Parra S, Alcaraz LD, Guzmán P (2006). The ATL gene family from Arabidopsis thaliana and Oryza sativa comprises a large number of putative ubiquitin ligases of the RING-H2 type. J. Mol. Evol..

[85] Sadanandom A, Bailey M, Ewan R, Lee J, Nelis S (2012). The ubiquitin–proteasome system: Central modifier of plant signalling. New Phytol..

[86] Duplan V, Rivas S (2014). E3 ubiquitin-ligases and their target proteins during the regulation of plant innate immunity. Front. Plant Sci..

[87] Kelley DR (2018). E3 Ubiquitin ligases: key regulators of hormone signaling in plants. Mol. Cell. Proteomics.

[88] Mandal A, Sharma N, Muthamilarasan M, Prasad M (2018). Ubiquitination: a tool for plant adaptation to changing environments. Nucl..

[89] Echevarría-Zomeño S (2016). Dissecting the proteome dynamics of the early heat stress response leading to plant survival or death in Arabidopsis. Plant. Cell Environ..

[90] You Q (2016). An E3 ubiquitin ligase-BAG protein module controls plant innate immunity and broad-spectrum disease resistance. Cell Host Microbe.

[91] Li Y, Kabbage M, Liu W, Dickman MB (2016). Aspartyl protease-mediated cleavage of BAG6 is necessary for autophagy and fungal resistance in plants. Plant Cell.

[92] Kabbage M, Kessens R, Bartholomay LC, Williams B (2017). The life and death of a plant cell. Annu. Rev. Plant Biol..

[93] Vo KTX (2015). Molecular insights into the function of ankyrin proteins in plants. J. Plant Biol..

[94] Bastien M, Sonah H, Belzile F (2014). Genome wide association mapping of Sclerotinia sclerotiorum resistance in soybean with a genotyping-by-sequencing approach. Plant Genome.

[95] Liu S, Ge F, Huang W, Lightfoot DA, Peng D (2019). Effective identification of soybean candidate genes involved in resistance to soybean cyst nematode via direct whole genome re-sequencing of two segregating mutants. Theor. Appl. Genet..

[96] Hunziker P (2020). De novo indol-3-ylmethyl glucosinolate biosynthesis, and not long-distance transport, contributes to defence of Arabidopsis against powdery mildew. Plant. Cell Environ..

[97] Bednarek P (2009). A glucosinolate metabolism pathway in living plant cells mediates broad-spectrum antifungal defense. Science.

[98] González-Lamothe R (2009). Plant antimicrobial agents and their effects on plant and human pathogens. Int. J. Mol. Sci..

[99] Chen J (2020). The phytopathogenic fungus Sclerotinia sclerotiorum detoxifies plant glucosinolate hydrolysis products via an isothiocyanate hydrolase. Nat. Commun..

[100] Sekhwal M (2015). Disease resistance gene analogs (RGAs) in plants. Int. J. Mol. Sci..

[101] Chisholm ST, Coaker G, Day B, Staskawicz BJ (2006). Host-microbe interactions: Shaping the evolution of the plant immune response. Cell.

[102] Fukuda WMG, Silva SO, Iglesias C (2002). Cassava Breeding. *Crop. Breed. Appl*. Biotechnol..

[103] Phillips JM, Hayman DS (1970). Improved procedures for clearing roots and staining parasitic and vesicular-arbuscular mycorrhizal fungi for rapid assessment of infection. Trans. Br. Mycol. Soc..

[104] Doyle JJ, Doyle JL (1990). Isolation of plant DNA from fresh tissue. Focus.

[105] Ferreira CF (2019). Brief note rapid plant DNA and RNA extraction protocol using a bench drill. Genet. Mol. Res..

[106] Klepikova AV, Logacheva MD, Dmitriev SE, Penin AA (2015). RNA-seq analysis of an apical meristem time series reveals a critical point in Arabidopsis thaliana flower initiation. BMC Genom..

[107] Soni P (2020). Transcriptome analysis identified coordinated control of key pathways regulating cellular physiology and metabolism upon Aspergillus flavus infection resulting in reduced aflatoxin production in groundnut. J. Fungi.

[108] Ghamari R, Ahmadikhah A, Tohidfar M, Bakhtiarizadeh MR (2022). RNA-Seq analysis of Magnaporthe grisea transcriptome reveals the high potential of ZnO nanoparticles as a nanofungicide. Front. Plant Sci..

[109] R Core Team. *A Language and Environment for Statistical Computing* (2020).

[110] Lun ATL, Chen Y, Smyth GK (2016). It’s DE-licious: A recipe for differential expression analyses of RNA-seq experiments using quasi-likelihood methods in edgeR. Methods Mol. Biol..

[111] Benjamini Y, Hochberg Y (1995). Controlling the false discovery rate: A practical and powerful approach to multiple testing. J. R. Stat. Soc. Ser. B.

[112] Conesa A (2005). Blast2GO: A universal tool for annotation, visualization and analysis in functional genomics research. Bioinformatics.

[113] Thimm O (2004). mapman: A user-driven tool to display genomics data sets onto diagrams of metabolic pathways and other biological processes. Plant J..

[114] Lohse M (2014). Mercator: a fast and simple web server for genome scale functional annotation of plant sequence data. Plant. Cell Environ..

[115] UniProt Consortium (2015). UniProt: A hub for protein information. Nucleic Acids Res..

[116] Moreno I, Gruissem W, Vanderschuren H (2011). Reference genes for reliable potyvirus quantitation in cassava and analysis of Cassava brown streak virus load in host varieties. J. Virol. Methods.

