# Supplementary material for: Comparative analysis of infected cassava root transcriptomics reveals candidate genes for root rot disease resistance
Source: Sci Rep. 2024 May 8;14:10587. doi: 10.1038/s41598-024-60847-4 (PMC11078935; doi:10.1038/s41598-024-60847-4)
Supplement: Supplementary file 1 — Supplementary Tables. [file 41598_2024_60847_MOESM1_ESM.docx]

**Comparative analysis of infected cassava root transcriptomics reveals candidate genes for root rot disease resistance**

Table S1. Description of the total number of sequenced, filtered and alignment fragments for each library constructed for analysis of the transcriptome of contracting cassava genotypes for root rot tolerance.

| **Library** | **Reads** | | | | | | |
| --- | --- | --- | --- | --- | --- | --- | --- |
|  | **Replication** | **Total** | **Exonics** | **Intronics** | **Intergenics** | **Aligned** | |
| BGM 1345 - Healthy Initial | 1 | 47.460.062 | 71.10% | 18.50% | 10.40% | 23.451.517 | 49.41% |
|  | 2 | 49.479.248 | 71.60% | 18.70% | 9.70% | 26.813.088 | 54.19% |
| BGM 1345 - Infested Initial | 1 | 48.386.335 | 72.20% | 17.70% | 10% | 20.677.745 | 42.74% |
|  | 2 | 48.711.663 | 71.50% | 18.20% | 10.20% | 20.779.424 | 42.66% |
| BRS Kiriris - Healthy Initial | 1 | 50.647.475 | 68.30% | 18.70% | 12.90% | 29.052.993 | 57.37% |
|  | 2 | 51.507.977 | 68.40% | 19.10% | 12.50% | 28.211.967 | 54.77% |
| BRS Kiriris - Infested Initial | 1 | 46.289.890 | 69.80% | 17.60% | 12.60% | 24.274.005 | 52.44% |
|  | 2 | 48.647.987 | 70.80% | 17.10% | 12% | 26.115.025 | 53.68% |
| BGM 1345 - Healthy Final | 1 | 47.166.150 | 63.80% | 22.70% | 13.40% | 13.534.249 | 28.70% |
|  | 2 | 51.223.120 | 69.60% | 18.70% | 11.60% | 22.760.961 | 44.44% |
| BGM 1345 - Infested Final | 1 | 53.351.758 | 67.90% | 20% | 12% | 21.325.645 | 39.97% |
|  | 2 | 50.152.538 | 67.50% | 19.50% | 12.90% | 18.983.179 | 37.85% |
| BRS Kiriris - Healthy Final | 1 | 49.855.411 | 71.20% | 15.90% | 12.90% | 25.622.057 | 51.39% |
|  | 2 | 50.250.629 | 73.10% | 15.40% | 11.40% | 27.548.695 | 54.82% |
| BRS Kiriris - Infested Final | 1 | 50.874.740 | 67.50% | 18.60% | 13.90% | 26.206.987 | 51.51% |
|  | 2 | 51.877.379 | 66.80% | 19% | 14.20% | 26.711.602 | 51.49% |
| BGM-1345 - Control | 1 | 53.575.090 | 73.90% | 17.20% | 9% | 30.826.552 | 57.55% |
|  | 2 | 52.640.163 | 64.70% | 23.40% | 11.90% | 28.133.110 | 53.45% |
| BRS Kiriris - Control | 1 | 56.040.228 | 69.60% | 18.80% | 11.60% | 35.479.598 | 63.32% |
|  | 2 | 54.802.454 | 68% | 19.70% | 12.30% | 33.710.101 | 61.52% |
| Total |  | 1.012.940.297 |  |  |  | 510.218.500 |  |

Table S2. List of fungal isolates

| **Isolates** | **Genus** | **Root rot complex** |  | **Isolates** | **Genus** | **Root rot complex** |
| --- | --- | --- | --- | --- | --- | --- |
| 01.SE | *Fusarium* | Dry rot |  | A11.4 | *Fusarium* | Dry rot |
| 02.SE | *Fusarium* | Dry rot |  | A13.2 | *Fusarium* | Dry rot |
| 03.SE | *Fusarium* | Dry rot |  | A13.3 | *Fusarium* | Dry rot |
| 04.SE | *Lasiodiplodia* | Black rot |  | A13.4 | *Fusarium* | Dry rot |
| 05.SE | *Fusarium* | Dry rot |  | A2.5 | *Fusarium* | Dry rot |
| 07.SE | *Fusarium* | Dry rot |  | A4 | *Lasiodiplodia* | Black rot |
| 09.SE | *Fusarium* | Dry rot |  | A4.1 | *Fusarium* | Dry rot |
| 11.SE | *Fusarium* | Dry rot |  | A4.2 | *Fusarium* | Dry rot |
| 12.SE | *Fusarium* | Dry rot |  | A4.4 | *Fusarium* | Dry rot |
| 13.SE | *Fusarium* | Dry rot |  | A4.5 | *Fusarium* | Dry rot |
| 14.SE | *Fusarium* | Dry rot |  | A6 | *Fusarium* | Dry rot |
| 16.SE | *Fusarium* | Dry rot |  | A6.2 | *Fusarium* | Dry rot |
| 18.SE | *Fusarium* | Dry rot |  | A6.3 | *Fusarium* | Dry rot |
| 22.SE | *Fusarium* | Dry rot |  | A7.1 | *Fusarium* | Dry rot |
| 23.SE | *Fusarium* | Dry rot |  | A7.3 | *Fusarium* | Dry rot |
| 26.SE | *Fusarium* | Dry rot |  | A7.6 | *Fusarium* | Dry rot |
| 29.SE | *Fusarium* | Dry rot |  | A7.7 | *Fusarium* | Dry rot |
| 30.SE | *Lasiodiplodia* | Black rot |  | A7.8 | *Fusarium* | Dry rot |
| 31.SE | *Lasiodiplodia* | Black rot |  | A8.3 | *Fusarium* | Dry rot |
| 32.SE | *Fusarium* | Dry rot |  | A8.5 | *Fusarium* | Dry rot |
| 33.SE | *Fusarium* | Dry rot |  | A8.6 | *Fusarium* | Dry rot |
| 34.SE | *Fusarium* | Dry rot |  | A9.1 | *Fusarium* | Dry rot |
| 35.SE | *Fusarium* | Dry rot |  | A9.3 | *Fusarium* | Dry rot |
| 36.SE | *Lasiodiplodia* | Black rot |  | A8.2 | *Fusarium* | Dry rot |
| 37.SE | *Lasiodiplodia* | Black rot |  | A9 | *Fusarium* | Dry rot |
| A10.1 | *Fusarium* | Dry rot |  | A11.3 | *Fusarium* | Dry rot |
| A10.2 | *Fusarium* | Dry rot |  | A13 | *Fusarium* | Dry rot |
| A10.3 | *Fusarium* | Dry rot |  | 24.SE | *Fusarium* | Dry rot |
| A11.1 | *Fusarium* | Dry rot |  | 28.SE | *Fusarium* | Dry rot |
| A11.2 | *Fusarium* | Dry rot |  | 38.SE | *Fusarium* | Dry rot |
