# Supplementary material for: Comparative analysis of infected cassava root transcriptomics reveals candidate genes for root rot disease resistance
Source: Sci Rep. 2024 May 8;14:10587. doi: 10.1038/s41598-024-60847-4 (PMC11078935; doi:10.1038/s41598-024-60847-4)
Supplement: Supplementary file 2 — Supplementary Figure S1. [file 41598_2024_60847_MOESM2_ESM.docx]

**Comparative analysis of infected cassava root transcriptomics reveals candidate genes for root rot disease resistance**


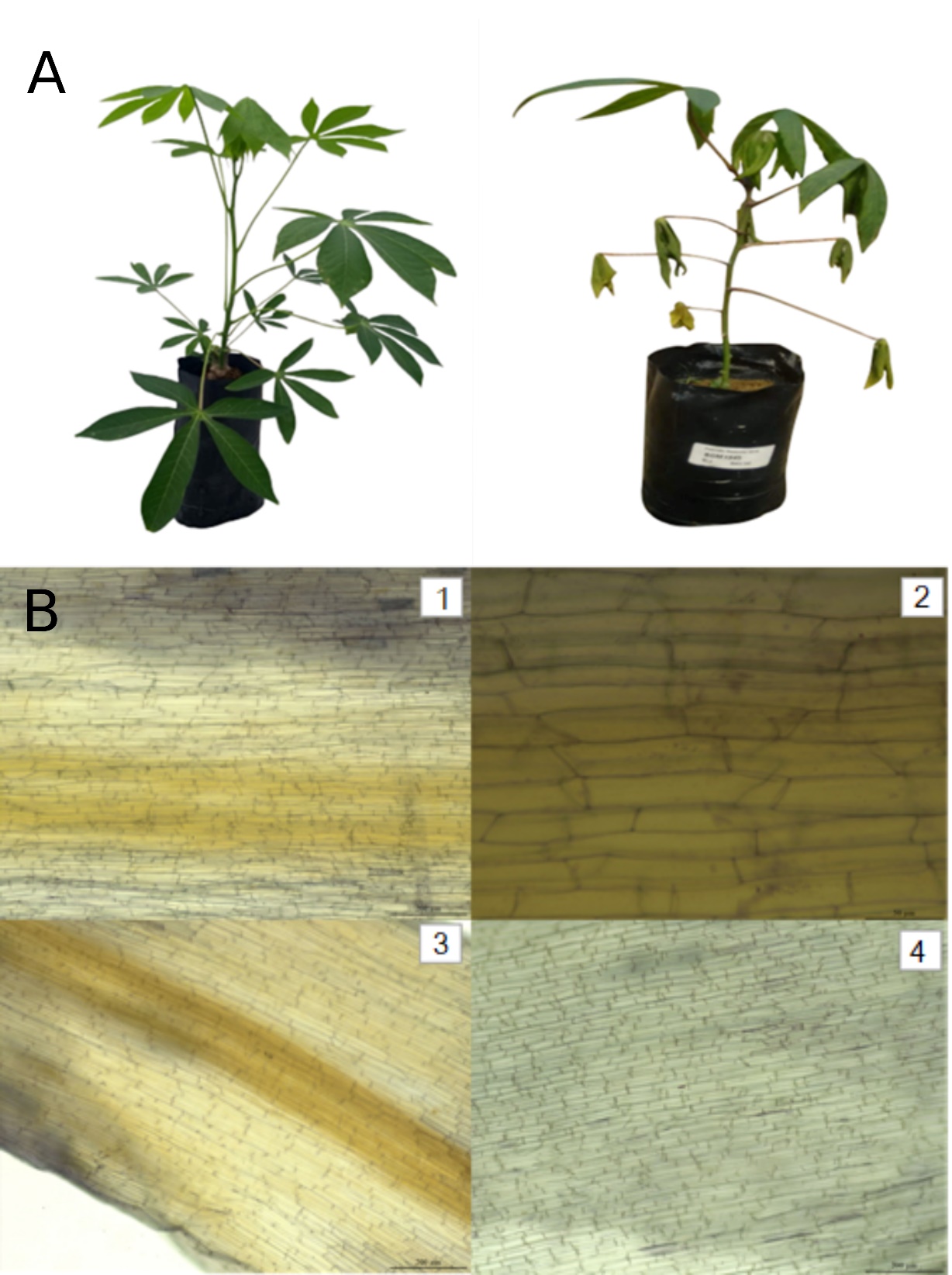


**Figure S1**. Cassava plants subjected to infection with root rot-causing pathogens. A – left: cultivar BRS Kiriris expressing no foliar symptoms; right: BGM-1345 expressing wilt symptoms on leaves. B Clarification of root fragments (control) without the presence of fungal structures.
